# *Drosophila* H2Av negatively regulates the activity of the IMD pathway via facilitating Relish SUMOylation

**DOI:** 10.1371/journal.pgen.1009718

**Published:** 2021-08-09

**Authors:** Ruijuan Tang, Wuren Huang, Jingmin Guan, Qiuning Liu, Brenda T. Beerntsen, Erjun Ling

**Affiliations:** 1 University of Chinese Academy of Sciences, Beijing, China; 2 Key Laboratory of Insect Developmental and Evolutionary Biology, CAS Center for Excellence in Molecular Plant Sciences, Shanghai Institute of Plant Physiology and Ecology, Chinese Academy of Sciences, Shanghai, China; 3 Jiangsu Key Laboratory for Bioresources of Saline Soils, Jiangsu Synthetic Innovation Center for Coastal Bio-agriculture, Jiangsu Provincial Key Laboratory of Coastal Wetland Bioresources and Environmental Protection, School of Wetland, Yancheng Teachers University, Yancheng, China; 4 Veterinary Pathobiology, University of Missouri, Columbia, Missouri, United States of America; 5 State Key Laboratory of Plant Genomics, Institute of Genetics and Developmental Biology, The Innovative Academy of Seed Design, Chinese Academy of Sciences, Beijing, China; George Washington University, UNITED STATES

## Abstract

Insects depend on the innate immune response for defense against a wide array of pathogens. Central to *Drosophila* immunity are antimicrobial peptides (AMPs), released into circulation when pathogens trigger either of the two widely studied signal pathways, Toll or IMD. The Toll pathway responds to infection by Gram-positive bacteria and fungi while the IMD pathway is activated by Gram-negative bacteria. During activation of the IMD pathway, the NF-κB-like transcription factor Relish is phosphorylated and then cleaved, which is crucial for IMD-dependent AMP gene induction. Here we show that loss-of-function mutants of the unconventional histone variant H2Av upregulate IMD-dependent AMP gene induction in germ-free *Drosophila* larvae and adults. After careful dissection of the IMD pathway, we found that Relish has an epistatic relationship with H2Av. In the H2Av mutant larvae, SUMOylation is down-regulated, suggesting a possible role of SUMOylation in the immune phenotype. Eventually we demonstrated that Relish is mostly SUMOylated on amino acid K823. Loss of the potential SUMOylation site leads to significant auto-activation of Relish *in vivo*. Further work indicated that H2Av regulates Relish SUMOylation after physically interacting with Su(var)2-10, the E3 component of the SUMOylation pathway. Biochemical analysis suggested that SUMOylation of Relish prevents its cleavage and activation. Our findings suggest a new mechanism by which H2Av can negatively regulate, and thus prevent spontaneous activation of IMD-dependent AMP production, through facilitating SUMOylation of the NF-κB like transcription factor Relish.

## Author summary

Toll and IMD signaling pathways should be involved in the production of antimicrobial peptides in animals upon infection. Immunity responses are energy consuming. Thus, these two pathways are fine-tuned. Animal H2A variant histones are involved in many physiological functions. In *Drosophila*, the production of antibacterial peptides is out of control in the mutant of H2A variant (*H2Av*^*810*^). After careful examination, we found that Relish, the transcription factor of the IMD pathway, was activated in this mutant. Eventually we demonstrate that Relish can be SUMOylated with the involvement of H2Av. Loss of the main SUMOylation site in Relish induces it to auto-activate following over-expression. Therefore, H2Av is a negative regulator of the IMD signaling pathway by maintaining the normal level of Relish SUMOylation in *Drosophila*.

## Introduction

The fruit fly *Drosophila melanogaster* and other species of insects live on foods enriched with diverse micro-organisms like bacteria, fungi, parasites and viruses. Most microbes are pathogenic if they reach the internal milieu through the spiracles or wounds in the epidermis [1]. In an evolutionary arms race, *Drosophila* and other insects have evolved highly efficient humoral and cellular immunity against their natural insect pathogens [2–4]. Cellular immunity is immediate, and mediated by circulating haemocytes that phagocytose small microbes and encapsulate large parasites upon recognition of the invading pathogens [4]. Haemocytes are also involved in coagulation and melanization, which are important mechanisms of cellular immunity [4]. Humoral immunity, such as induction of antimicrobial peptide (AMP) gene expression, is slower to deploy and takes several hours after infection [2–5]. Upon detection of invading pathogens, the transcription of specific AMP genes is triggered by two NF-κB signaling pathways named the immune deficiency (IMD) and Toll pathways [2–5]. The Toll pathway is predominantly activated by fungi and Gram-positive bacteria [4], while the IMD pathway mainly detects Gram-negative bacteria [4,6]. Both pathways have specific NF-kB like transcription factors: Dorsal and Dif for the Toll pathway, and Relish for the IMD pathway [2–5]. Upon activation, these transcription factors undergo post-translational modifications, which are either released from their inhibitors or by the cleavage of an inhibitory fragment, preparing for nuclear translocation. Using the powerful genetics of *Drosophila*, we now understand the many positive and negative regulatory mechanisms that fine tune the activity of these two pathways [7,8].

SUMOylation is widely conserved in eukaryotes and regulates a wide variety of cellular and developmental processes [9,10]. Recent work indicates that SUMOylation is also involved in the regulation of immune activities [11]. *Drosophila* carries one gene encoding the SUMO homolog, named Smt3. Smt3 is activated by the ubiquitin-like protease (Ulp1), which cleaves the C-terminal extension from the immature Smt3 to expose a Gly-Gly motif [9]. The E1 activating enzyme Aos1/Uba2 and E2 conjugating enzyme Ubc9 are required for SUMO binding to a target protein. Eventually the E3 SUMO ligase Su(var)2-10 transfers Smt3 from E2 to the target protein to complete the SUMOylation. Ulp1 may also catalyze the removal of Smt3 from the target protein to reverse SUMOylation [9]. Therefore, SUMOylation may widely regulate *Drosophila* immune responses. Indeed, in *Drosophila*, Smt3 was shown to conjugate to the Toll pathway transcription factor Dorsal [12]. During the process of Smt3 conjugation to Dorsal, the E2 enzyme Ubc9 counteracts the Cactus-mediated inhibition of Dorsal nuclear localization [12]. In S2 cells, nuclear localization of a Dorsal-GFP fusion protein was enhanced in the presence of Cactus and Ubc9 [13]. In *Ubc9* mutants, the basement membrane of fat bodies loses its integrity, causing circulating hemocytes to infiltrate and aggregate into melanotic tumors [14]. Degringolade (Dgrn) is a SUMO-targeted ubiquitin ligase that connects the ubiquitin and SUMO pathways [15]. Dgrn is essential for *Drosophila* embryonic development [16]. A recent work demonstrates that *dgrn*^*DK*^ null mutant adults are susceptible to bacterial and fungal infections due to their inability to transcript Toll- and IMD-dependent AMPs [15]. Co-expression of *Dgrn* with either *PGRP-LCa* or the truncated Toll receptor (*Toll*^*△LRR*^) can constitutively activate the IMD or Toll pathway enhanced production of AMPs, indicating that Dgrn is involved in the systemic immune response. Further works demonstrate that Dgrn regulates Toll pathway activity by alleviating the inhibitory impact of Cactus and Groucho. Interestingly, another study shows that β-arrestin Kurtz (Krz) can control Toll signaling since knockdown of Krz upregulated the production of Drosomycin (Drs) [17]. Eventually Ulp1, a SUMO protease with desumoylating activity, was shown to interact with Krz. As a cofactor, Krz synergistically controls the SUMOylation of Dorsal and other factors with Ulp1 to maintain Toll pathway activity [17]. Loss of Krz and/or Ulp1 will imbalance SUMOylation and desumoylation *in vivo* and cause an inappropriate inflammatory response in the absence of pathogens. Therefore, the SUMOylation pathway and its components directly or indirectly affect humoral and cellular immunity in *Drosophila*.

Conversely, we know very little whether the IMD pathway is similarly regulated by SUMOylation. In the IMD pathway, the IκB Kinase (IKK) complex, IKKβ (also called ird5) and IKKγ (Kenny, Key), activates the NF-κB family member Relish (Rel) for cleavage by the caspase Dredd. Dredd cleavage releases the nuclear translocation fragment Rel-68 from its inhibitory fragment Rel-49 [18,19]. A recent study indicated that IKKβ was SUMOylated during bacterial challenge [20]. K152 of IKKβ was predicted to be the potential SUMOylation site and was then mutated. Western blot assays showed that the amount of SUMOylated ird5(K152A) mutant was lower than wild type ird5 [20]. Over-expression of the mutant ird5(K152A) protein led to significantly lower Attacin A expression than wild type ird5 [20]. This suggests that SUMOylation can also directly regulate IMD pathway activity through the kinase IKKβ. However, SUMOylation generally acts on transcription factors to regulate their transcriptional activity, and ird5 is not the transcription factor of the IMD pathway [20]. In analogy with SUMOylation of the Toll transcription factor Dorsal (Bhaskar et al., 2002), amino acid K313 of Relish was predicted to be the potential SUMOylation site [12], but this hypothesis remains to be experimentally proven. Thus, we still do not know whether the IMD transcription factor Relish can be SUMOylated to regulate its own activity.

Mammals harbor multiple H2A variant histones such as H2AZ and H2AX [21]. H2AZ is involved in transcriptional activation and maintenance of gene silencing [22], and H2AX plays an important role in DNA repair [23]. *Drosophila* has a single histone H2A variant named H2Av [24]. In *H2Av*^*810*^/*H2Av*^*810*^ null mutant larvae, the typical phenotypes are production of melanotic tumors and increased hematopoiesis, suggesting some form of immune dysfunction [25]. In this study, we investigated whether the immune phenotype of H2Av mutants might involve SUMOylation of immune pathway components. We found that H2Av can indeed regulate Relish SUMOylation after interacting with the E3 enzyme Su(var)2-10. Loss of the putative SUMOylation amino acid K823 in Relish enhanced its activation. Correspondingly, AMP genes were upregulated *in vivo* in the absence of extrinsic pathway activation. We therefore show that maintenance of Relish SUMOylation is an important mechanism to regulate IMD pathway activity *in vivo*. In this mechanism, H2Av is involved in Relish SUMOylation via interaction with Su(var)2-10.

## Results

### Loss of H2Av enhances the expression of antimicrobial peptides in *Drosophila* larvae and adults

**Fig 1:**
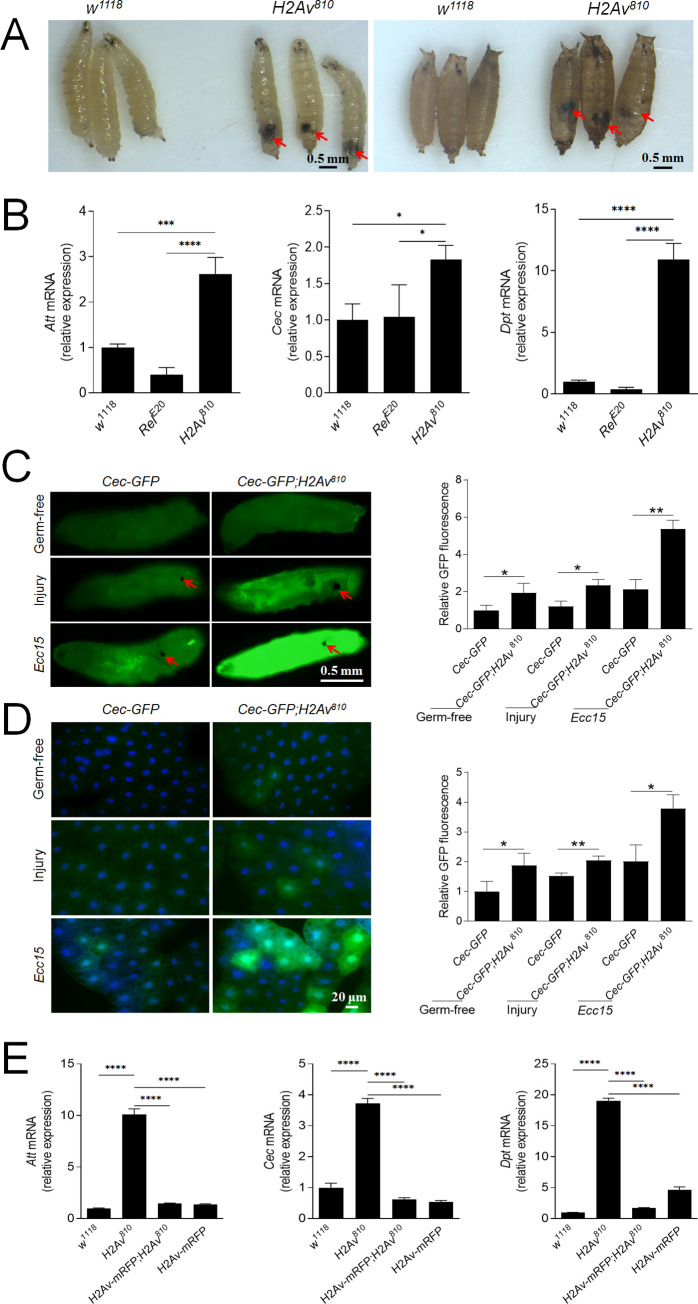
Loss of H2Av enhances AMP production. (A) Comparison of the morphology between wild type (*w*^*1118*^) and homozygous *H2Av*^*810*^/*H2Av*^*810*^ (named *H2Av*^*810*^) mutants of third instar larvae and pupae. Melanotic tumors as indicated by arrows appear near the posterior end of larvae and pupae. (B) Upregulation of AMPs in fat bodies of germ-free *H2Av*^*810*^ mutant larvae. A mutant of the IMD pathway transcription factor Relish (*Rel*^*E20*^) was used as a negative control. qPCR was done on dissected fat bodies from germ-free second-instar larvae. Data represent the average of at least three independent assays (mean ± SE). (C) Production of Cecropin was analyzed using the *Cec-GFP* reporter line. Germ-free second-instar larvae received injury or *Ecc15* immune challenge and GFP fluorescence was observed at 6 h. The arrows indicate the wounds that were melanized after injury. For quantification of GFP signal, uninfected *Cec-GFP* = 1. Mean values are presented ± SE (n = 3). (D) Production of Cecropin in fat bodies. Fat bodies of larvae as shown in (C) were observed and GFP fluorescence was quantified. Mean values are presented ± SE. (E) Expression of H2Av-mRFP to rescue the phenotype of up-regulation of AMP in the *H2Av*^*810*^ mutant. H2Av-mRFP was driven by the H2Av promoter. qPCR analysis of AMPs in fat bodies dissected from second-instar larvae. Data represent the average of at least three independent assays (mean ± SE). One way ANOVA with Tukey’s multiple comparisons test (B, E) or two-tailed Student’s t-test (C, D) was performed. *p < 0.05, **p < 0.01, ***p < 0.001, and ****p < 0.0001. Bar: (A, C) 0.5 mm; (D) 20 μm.

*Drosophila* has one H2Av variant, H2Av, that combines the features of mammalian H2A.Z and H2A.X [24]. *H2Av*^*810*^ is a null allele containing a 311 bp deletion that removes the second exon [26]. *H2Av*^*810*^ homozygous mutant larvae and pupae exhibited melanotic tumors near the posterior end (Fig 1A). In *H2Av*^*810*^ mutant larvae, there were significantly greater number of lamellocytes produced (S1A and S1B Fig), and this phenotype was partially rescued when the *H2Av*^*810*^ mutant was crossed with *H2Av-mRFP* (S1B Fig). In addition, PPO3 was expressed with or without immune challenge in the *H2Av*^*810*^ mutant (S1C Fig). PPO3 has phenoloxidase (PO) activity even without activation [27,28]. The production of lamellocytes and expression of PPO3 may partially contribute to the production of melanotic tumors [28]. Despite the presence of melanotic tumors, all *H2Av*^*810*^ larvae spent approximately two days longer than *w*^*1118*^ larvae to develop into pupae but failed to emerge. Preliminary work showed that *H2Av*^*810*^ larvae expressed higher antimicrobial peptide levels (AMP) at the early 3rd instar in comparison to wild type *w*^*1118*^. First, we assessed whether the AMP upregulation was due to the presence of microbiota. Germ free larvae of the wild type strain *w*^*1118*^ and Relish deficient strain *Rel*^*E20*^ were sampled before the wandering stage. *H2Av*^*810*^ larvae were also sampled at the same time point. The expression of IMD-dependent target genes *Diptericin* (*Dpt*), *Cecropin* (*Cec*) and *Attacin* (*Att*) was significantly enhanced in *H2Av*^*810*^ compared to *w*^*1118*^ and *Rel*^*E20*^ mutant larvae (Fig 1B). *Ecdysone 20-monooxygenase* that encodes the terminal gene to produce 20-hydroxyecdysone (20E) in the pathway of ecdysteroid production [29,30] was significantly down-regulated in *H2Av*^*810*^ larvae (S2A Fig). This indicates that in *H2Av*^*810*^ larvae AMP production is constitutively upregulated, independent of microbiota or the rising titer of 20E that usually takes place before the wandering stage [31,32]. To confirm that the loss of H2Av enhances the expression of AMP genes, wild-type and *H2Av*^*810*^ larvae carrying the reporter gene *Cec-GFP* (germ-free) were injured or infected by pricking with a needle dipped in a concentrated solution of Gram-negative bacteria *Erwinia carotovora carotovora 15* (*Ecc15*) [33]. At 6 h post infection, the GFP fluorescence of whole bodies (Fig 1C) and fat bodies (Fig 1D) was imaged and quantified. Lack of H2Av significantly enhanced the expression of *CecA* in the naive germ-free larvae and in those that received a clean injury or *Ecc15* challenge at 6 h (Fig 1C and 1D). The impact of H2Av on CecA expression was especially marked when compared to the clean injury and bacteria challenged larvae.

To confirm that the loss of H2Av can enhance the production of AMPs, we analyzed the impact of H2Av silencing *in vitro* and *in vivo* using *RNAi*. For knockdown experiments in S2 cells, silencing of Kenny (key), a gene encoding a component of the IKK complex, which activates the transcription factor Relish (Rel), was used as a negative control. S2 cells were stimulated with DAP-type peptidoglycan (PGN) to induce AMP expression. Knockdown of *H2Av* significantly enhanced the production of Att, Cec and Dpt compared to controls, whereas knockdown of *key* completely abolished IMD pathway activation (S3A Fig).

For knockdown experiments *in vivo*, three different RNAi constructs silencing *H2Av* were expressed with the ubiquitous, temperature-controlled driver *Act5C*^*ts*^. Expression of *Att*, *Cec* and *Dpt* was significantly up-regulated in the fat bodies of larvae (S4A Fig) and adults (S4C Fig). With each RNAi line, the expression of *H2Av* was also significantly knocked down in larvae (S4B Fig) and adults (S4D Fig). It is unclear why some AMPs are upregulated in one RNAi line but not in the others. When three RNAi lines of *mesh* were used for knocking down in the midgut EB or EC cells, RNAi3 did not significantly enhance the number of PH3-positive cells as the others following knockdown in EB cells [34]. Therefore, it seems that the phenomenon is not specific with H2Av RNAi although we do not know the exact reason. Of note, when *H2Av* was knocked down using the *Act-gal4* or *Tub-gal4* ubiquitous drivers, melanotic tumors appeared near the posterior end of the larvae (S5 Fig), similar to *H2Av*^*810*^ mutant larvae (Fig 1A). Therefore, loss of H2Av enhances the production of AMPs regardless of developmental stage.

In order to confirm that the loss of *H2Av* is responsible for the up-regulation of AMPs, the line of *H2Av-RFP* that expresses RFP-tagged functional His2Av in all cells under the control of His2Av [35] was expressed in *H2Av*^*810*^ mutants. Expression of H2Av-RFP in the *H2Av*^*810*^ larvae did not show any AMP upregulation as compared to the wild-type (Fig 1E), which indicates that the loss of H2Av impacts AMP production. In *H2Av*^*810*^ mutant larvae, *Drs* was also significantly up-regulated, which was not observed if H2Av-RFP was introduced in the mutant (S6A Fig). When *H2Av* was knocked down *in vivo*, *Drs* expression was also significantly enhanced (S6B Fig). All these data demonstrate that H2Av can negatively regulate AMP production by an unknown mechanism.

### Epistatic position of H2Av in the IMD pathway

**Fig 2:**
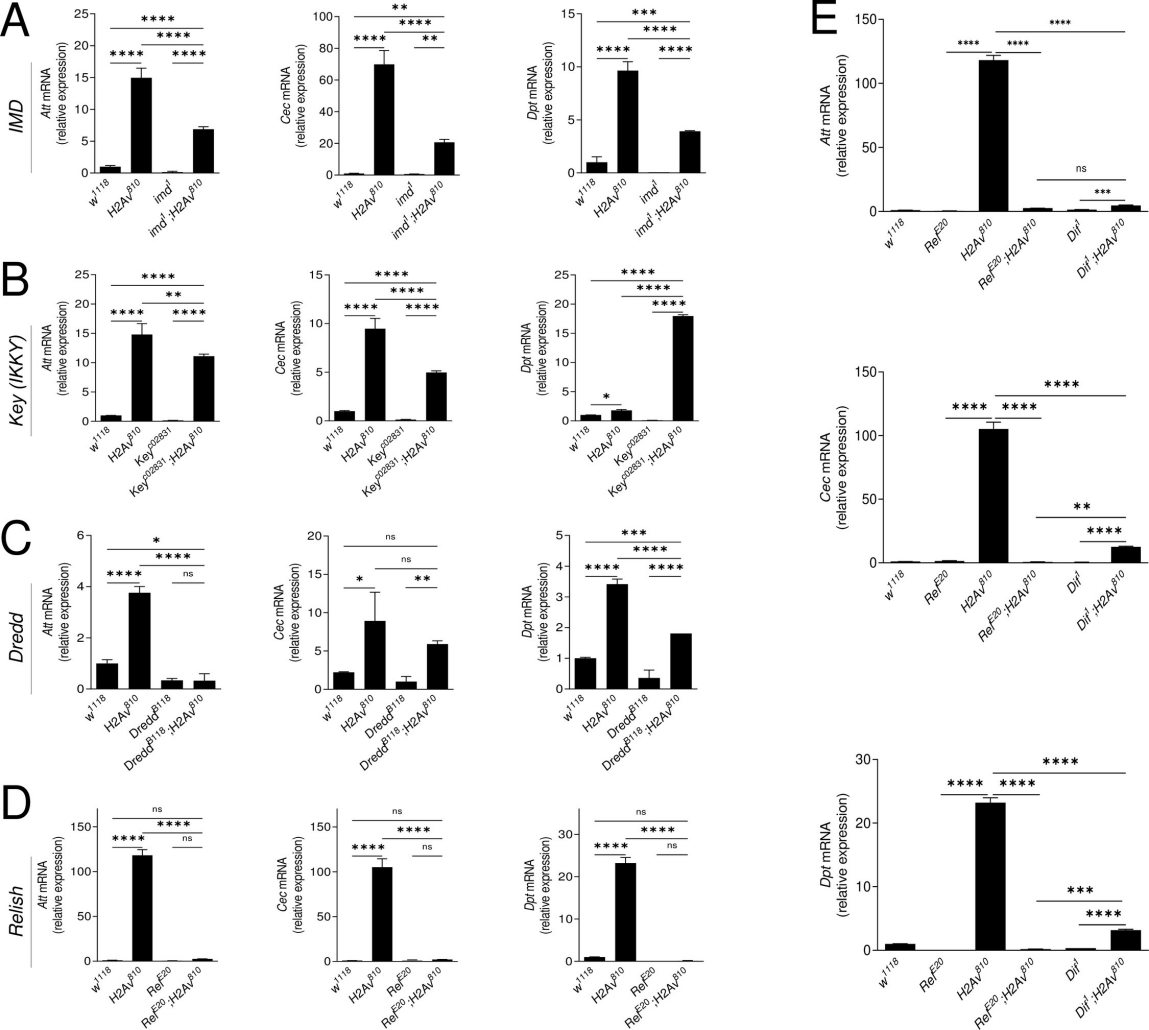
Epistatic relationship between H2Av and IMD pathway components. Homozygous *H2Av*^*810*^/*H2Av*^*810*^ flies were crossed with mutants of the main IMD pathway components (A, *imd*^*1*^; B, *Key*^*c02831*^; C, *Dredd*^*B118*^; D, *Rel*^*E20*^). A mutant of the Toll pathway component *Dif* (*Dif*^*1*^) was also crossed to analyze the relationship between Toll and H2Av (E). AMP transcription was assessed by qPCR on fat bodies of second-instar larvae. All genotypes are homozygous. Data represent the average of at least three independent assays (mean ± SE). One way ANOVA with Tukey’s multiple comparisons test was performed. *p < 0.05, **p < 0.01, ***p < 0.001, and ****p < 0.0001.

In *H2Av*^*810*^ mutant larvae, AMPs under both IMD pathway and Toll pathway control were dysregulated (Figs 1B–1D and S6A). Moreover, the Toll pathway genes *spz*, *tl*, *dorsal* and *dif* were all up-regulated (S6C Fig). In addition, the transcription factor Dorsal was detected in the nuclei of fat bodies of *H2Av*^*810*^ mutant larvae as observed in other mutants showing melanotic tumors [36] (S6D Fig). These observations indicate the expression of *Drs* in *H2Av*^*810*^ mutants. But further experiments are necessary to determine whether it is Toll-dependent since synergistic activation of both Toll and IMD pathways is important for *Drs* expression [2,37,38]. In addition, FOXO directly binds to the regulatory region of the *Drs* promoter in non-infected but starved animals [39]. The transcription of IMD pathway genes was also analyzed. Relish was significantly up-regulated in *H2Av*^*810*^ larvae that were conventionally reared (S7A Fig) or were reared under germ-free conditions (S7B Fig). The active fragment Rel-68 was detected in *H2Av*^*810*^ larval fat bodies using polyclonal antiserum against *Drosophila* Relish [40] but not in wild-type (S7C Fig). A commercial antibody against *Drosophila* Relish proven to be suitable for detecting Relish signal in nuclei after immuno-staining [41,42]. As shown in S7D Fig, Relish signal was detected in nuclei of fat bodies dissected from *w*^*1118*^ but not from *Rel*^*E20*^ after *Ecc15* immune challenge using this commercial antibody (S7D Fig). The immuno-staining of fat bodies indicated that a much higher signal of Relish was detected in whole cells and nuclei of *H2Av*^*810*^ larvae at the early 3rd instar (S7E Fig). Quantification of the signals in whole cells (S7F Fig) or nuclei (S7G Fig) showed that the amount of Relish was sufficiently higher in *H2Av*^*810*^ than in *w*^*1118*^. Having shown that *H2Av*^*810*^ can impact Relish cleavage, we therefore focused our interest on the influence of H2Av on the IMD pathway. To dissect the epistatic position of H2Av in the IMD pathway, mutants of several IMD pathway genes were combined with *H2Av*^*810*^ (Fig 2A–2D). The loss-of-function mutations *imd*^*1*^ (Fig 2A), *key*^*c02831*^ (Fig 2B) and *Dredd*^*B118*^ (Fig 2C) failed to abolish the production of AMPs induced by loss of H2Av. However, the Relish mutant *Rel*^*E20*^ rescued the *H2Av*^*810*^ phenotype (Fig 2D). Upon infection, the transcription factors, Relish (downstream in the IMD pathway) and Dif (downstream in the Toll pathway), synergistically regulate the production of various AMPs [2,37,38]. In order to understand whether Dif is also involved in regulating the expression of AMPs in the *H2Av*^*810*^ mutant, we crossed the mutant of Dif (*Dif*^*1*^) with *H2Av*^*810*^. Expression of *Att*, *Cec* and *Dpt* in mutants of *Rel*^*E20*^*;H2Av*^*810*^, *Dif*^*1*^;*H2Av*^*810*^ *and H2Av*^*810*^ were compared (Fig 2E). Although the expression of those AMPs in *Dif*^*1*^;*H2Av*^*810*^ and *Rel*^*E20*^*;H2Av*^*810*^ were decreased when compared with that of the *H2Av*^*810*^ mutant, AMPs in *Dif*^*1*^;*H2Av*^*810*^ were higher or significantly higher than that in *Rel*^*E20*^*;H2Av*^*810*^, indicating that Relish is likely more important than Dif in this mutant. akirin is a positive regulator of the IMD pathway that can increase activity of Rel in the nucleus [43,44]. In the *H2Av*^*810*^ mutant, akirin was significantly down-upregulate if compared with the wild type *w*^*1118*^ (S2B Fig). Therefore, akirin is not the reason for the enhancement of AMP production in the *H2Av*^*810*^ mutant. Different from other AMPs, the expression of Dpt was inhibited in the *Dredd*^*B118*^*;H2Av*^*810*^ mutant (Fig 2C). H2Av is an important histone variant that is directly or indirectly involved in the regulation of transcription and expression of many important genes [45]. We conclude that loss of *H2Av* is likely to up-regulate or down-regulate some genes including transcriptional factors, which may then bind to the Dpt promoter to influence its expression in the *H2Av*^*810*^ mutant. Taken together, all these data demonstrate that H2Av likely acts at the level of Relish to regulate the activity of the IMD pathway.

### Loss of H2Av decreases SUMOylation activity *in vivo*

**Fig 3:**
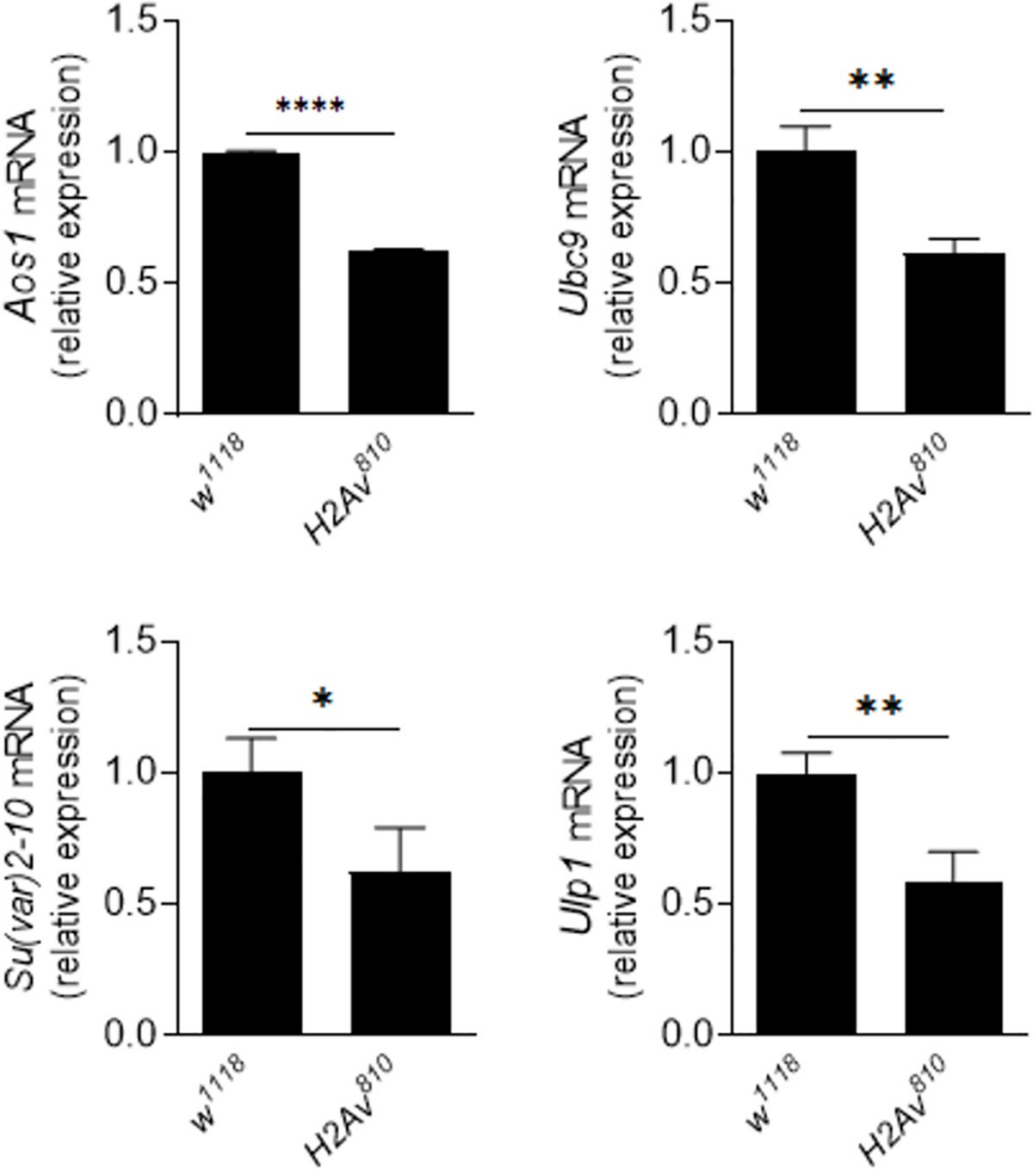
Loss of H2Av appears to decrease the activity of the SUMOylation pathway. qPCR analysis of components of the *Drosophila* SUMOylation pathway in fat bodies of *w*^*1118*^ and *H2Av*^*810*^ second-instar larvae. Data represent the average of at least three independent assays (mean ± SE). Two-tailed Student’s t-test was performed. *p < 0.05, **p < 0.01, ***p < 0.001, and ****p < 0.0001.

Ubc9, the E2 conjugating enzyme in the SUMOylation pathway in *Drosophila [46]*, accepts SUMO (Smt3) and transfers it to the target proteins [9]. Mutations in the *Drosophila Ubc9* (*dUbc9*) gene induce melanotic tumors near the posterior end of larvae, expression of several AMPs, and differentiation of lamellocytes [14,47–49]. In *H2Av*^*810*^ mutant larvae, many lamellocytes were also produced (S1 Fig). Given the phenotypic similarity of Ubc9 and H2Av mutants on hematopoiesis and lamellocyte differentiation, we wondered whether the SUMOylation pathway might be affected by the loss of H2Av. The *Drosophila* SUMOylation pathway consists of the heterodimeric El-activating enzyme Aos1-Uba2, the E2-conjugating enzyme Ubc9, and an E3 SUMO ligase Su(var)2-10 [9]. *Ulp1* encodes a cysteine protease that catalyzes SUMO maturation and SUMO deconjugation [9,17]. In *H2Av*^*810*^ mutant larvae, the expression of *Aos1* (E1), *Ubc9* (E2), *Su(var)2-10* (E3) and *Ulp1* were significantly down-regulated (Fig 3). When a polyclonal antibody against *Drosophila* SUMO was used for a Western blot, the amount of SUMOylated proteins decreased in the fat bodies of *H2Av*^*810*^ mutant larvae when compared with the control *w*^*1118*^ larvae (S8 Fig). These results show that absence of H2Av probably decreases the activity of the SUMOylation pathway in fat bodies.

### SUMOylation of Relish

**Fig 4:**
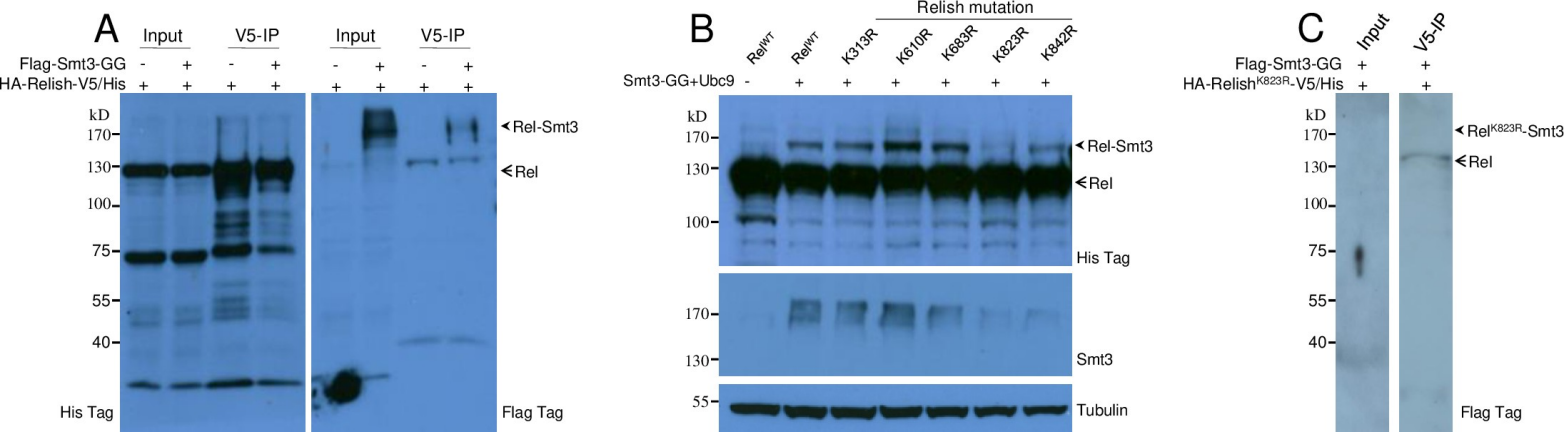
SUMOylation of Relish. (A) Relish can be SUMOylated. Wild type Relish (HA-Rel^WT^-V5/His) was expressed alone or co-expressed with Flag-Smt3-GG in S2 cells. Antibody against the V5-tag was applied for IP and enrichment. The arrowhead points to SUMOylated Relish (labeled Rel-Smt3); the arrow points to the position of Relish. (B) Identification of the main SUMOylation amino acid in Relish. The potential SUMOylation sites (lysine) were predicted online (http://sumosp.biocuckoo.org). Each lysine was mutated into arginine respectively. Rel^WT^ and each Relish mutant as indicated were co-expressed with Smt3-GG and Ubc9. The arrowhead points to the SUMOylated Relish (Rel-Smt3); the arrow indicates Relish proteins. Polyclonal antiserum against Smt3 was also applied to show the SUMOylated proteins. (C) The supposed Rel^K823R^-Smt3 cannot be enriched after IP. HA-Rel^K823R^-V5/His was co-expressed with Flag-Smt3-GG in S2 cells for IP and enrichment. The supposed Rel^K823R^-Smt3 was indicated based on molecular weight size.

In the larvae of the *Ubc9*^*-/-*^ mutant, several AMPs are up-regulated [14,47–49]. Although the nuclear localization of Dorsal and Dif were observed in this mutant [14,48], it is unknown whether nuclear Relish can be detected when the expression of Ubc9 is inhibited. When Ubc9 expression was knocked down, melanotic tumors were observed near the posterior end (S9A Fig). In addition, the transcription of Ubc9 and SUMOylated proteins were decreased after knockdown of Ubc9 (S9B Fig). Relish was detected in nuclei of fat bodies of Ubc9 knockdown larvae but not control and Ubc9 over-expression larvae (S9C Fig). Several AMPs were up-regulated when Ubc9 was knocked down (S9D Fig), indicating that SUMOylation may be negatively involved in the regulation of Relish activation. We suspected that Relish might be SUMOylated in *Drosophila*, a process that is likely influenced by loss of H2Av. Before activation and translocation into nuclei, Relish localizes to the cytoplasm. SUMOylation can also occur in the cytoplasm. H2Av primarily locates to the nucleus but a fraction remains in the cytoplasm (S10A Fig), which was confirmed by detection of rH2Av-FLAG in S2 cells and lamin in the nuclear fractions (S10C and S10D Fig). To confirm Relish SUMOylation *in vitro*, we co-expressed the *Drosophila* SUMO gene Smt3 with conserved Gly-Gly motif as the carboxyl end of the mature protein (Flag-Smt3-GG) and wild type Relish (*HA-Rel*^*WT*^*-V5/His*) containing different tags at the N- and C-terminus in S2 cells. Relish was immunoprecipitated using an antibody against the C-terminal V5-tag. The input cell lysate and V5-IP solution for Western blot were detected by an anti-His-Tag antibody (to reveal Rel^WT^) and an anti-Flag-tag antibody (to reveal Flag-Smt3-GG). The antibody against the Flag-tag revealed an additional band (~170 kD) after enrichment for Relish (Fig 4A). This result indicates that Rel^WT^ is likely a substrate of SUMOylation.

To confirm the SUMOylation of Relish, all the potential SUMOylated sites of Relish were predicted using the SUMOsp online tool (http://sumosp.biocuckoo.org) and each corresponding lysine (K) was mutated to arginine (R) respectively. Similar amounts of plasmids containing Rel^WT^ or different Relish mutants were separately co-expressed with plasmids containing Smt3-GG and Ubc9 in S2 cells. Rel^WT^ was clearly SUMOylated when co-expressed with Smt3-GG and Ubc9 compared to Rel^WT^ alone (Fig 4B). SUMOylated Rel was detected for all but one mutant with a modification at K823. And then *HA-Rel*^*WT*^*-V5/His* was over-expressed with *Flag-Smt3-GG* in S2 cells. After enrichment by V5-IP as Fig 4A, SUMOylated Rel^K823R^ was not detected (Fig 4C). This suggests that K823 was the main amino acid for SUMOylation. Detection by polyclonal antiserum against *Drosophila* Smt3 also confirmed that K823 was the main site for SUMOylation (Fig 4B).

Next, we determined whether Relish SUMOylation or decreased SUMOylation due to loss of the main SUMOylation site had any influence on Relish cleavage. Signal-dependent activation of Relish requires cleavage after residue D545 [19]. When D545 was mutated, mutant Rel^D545A^ over-expressed in S2 cells failed to be cleaved like Rel^WT^ if PGN was added (S11 Fig). Rel^D545A^ could be SUMOylated like Rel^WT^. However, after SUMOylation, Rel^WT^ but not Rel^D545A^ was cleaved when PGN was added to cells. When the main SUMOylation site was mutated (Rel^K823R^), SUMOylation was inhibited but cleavage was not affected. The double mutant Rel^D545A/K823R^ with cleavage site and main SUMOylation site mutated could neither be cleaved nor SUMOylated. In this study, to prevent Relish auto-cleavage after over-expression *in vitro* [18], cells were collected after transient transfection for 30 h (normally 48 h or even longer). Taken together, these data demonstrate that *Drosophila* Relish can be SUMOylated at K823 as the main site for its SUMOylation, and Relish SUMOylation and cleavage are independent.

### Loss of SUMOylation enhances Relish cleavage and AMP production *in vivo*

**Fig 5:**
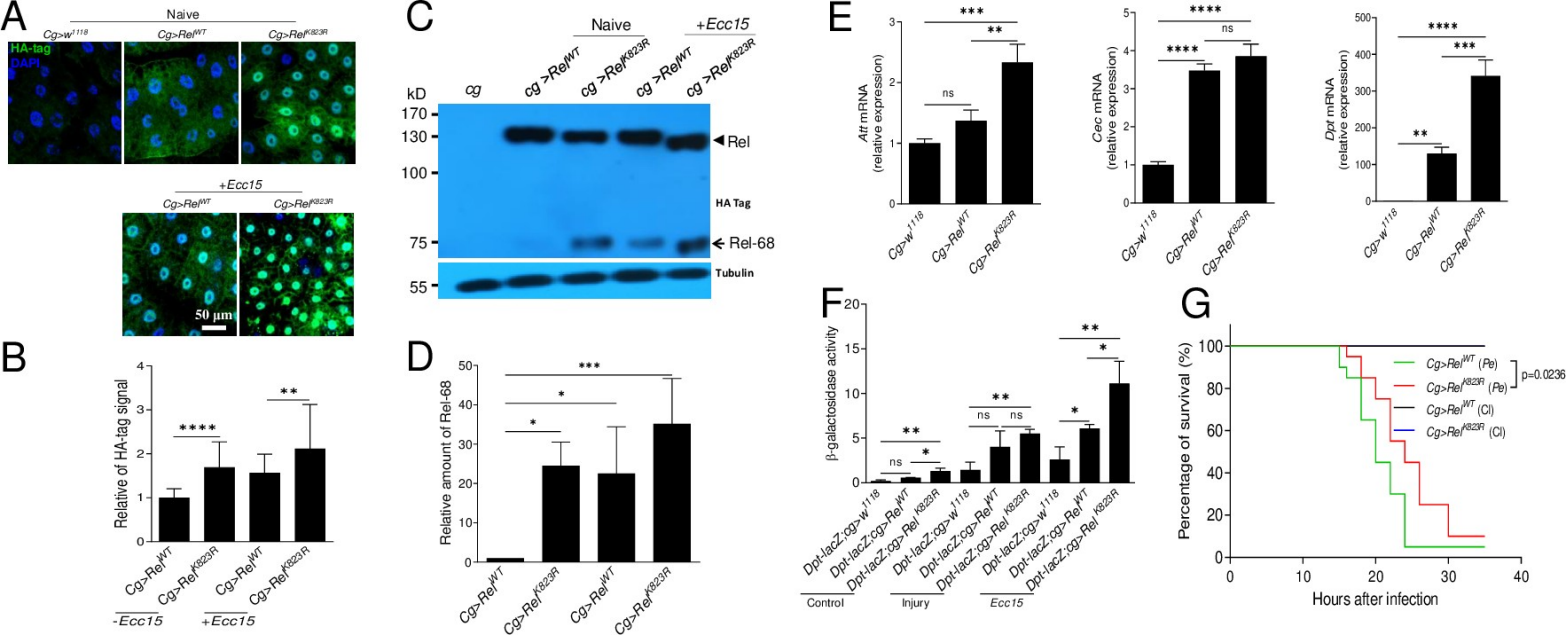
Loss of SUMOylation enhances Relish activation and AMP production *in vivo*. (A) Loss of the main potential SUMOylation amino acid enhances Relish nuclear translocation. *HA-Rel*^*WT*^*–V5/His* and *HA-Rel*^*K823R*^*-V5/His* (loss of SUMOylation) were over-expressed in fat bodies using the *Cg-gal4* driver. Fat bodies dissected from naïve larvae and larvae (early 3rd instar) challenged with *Ecc15* were immuno-stained using antibody against HA-tag. (B) Quantification of the HA-tag signal in the nuclei of fat bodies as shown in (A). For quantification, the HA-tag signal in the nuclei of *Cg>w*^*1118*^ = 1. Data represent the average of at least three independent assays (mean ± SE). (C) Detection of active Rel-68 in the fat bodies. As shown in (A), Rel^WT^ and Rel^K823R^ were separately over-expressed in fat bodies using Cg-gal4. Antibody against the HA-tag was applied to detect Relish (arrowhead) and Rel-68 (arrow). Rel-68 is the transcriptionally active fragment after cleavage. (D) Quantification of the band density of Rel-68 shown in (C). This experiment was repeated four times. The amount of Rel-68 produced from fat bodies of *Cg>Rel*^*WT*^ (-*Ecc15*) is equal to 1 for quantification. Mean values are presented ± SE. (E) qPCR analysis of AMP production in fat bodies with Rel^WT^ or Rel^K823R^ over-expressed *in vivo*. Data represent the average of at least three independent assays (mean ± SE). (F) The Dpt-lacZ reporter line was crossed with the indicated lines to over-express either Rel^WT^ or Rel^K823R^ in fat bodies. The above larvae (n = 20) were subjected to injury or *Ecc15* immune challenge, then dissected at 6 h to obtain fat bodies for a β-galactosidase activity assay. Data represent the average of at least three independent assays (mean ± SE). (G) Survival analysis after *Pseudomonas entomophila* (*Pe*) infection. The injury of a clean injection (CI) is the control. A log-rank analysis on the Kaplan-Meier data showed a statistically significant difference in survival between larvae over-expressing Rel^WT^ or Rel^K823R^ in fat bodies. One way ANOVA with Tukey’s multiple comparisons test (E, D, F) or two-tailed Student’s t-test (B) was performed. *p < 0.05, **p < 0.01, ***p < 0.001, and ****p < 0.0001. Bar: 50 μm.

To test the role of Relish SUMOylation *in vivo*, Upstream Activator Sequence (UAS) transgenes of *HA-Rel*^*WT*^*-V5/His* and *HA-Rel*^*K823R*^*-V5/His* (loss of potential SUMOylation site) were constructed. Rel^WT^ and Rel^K823R^ were over-expressed in fat bodies using Cg-gal4. To avoid the influence of 20E on the activation of the IMD pathway at the wandering stage [31,32], the fat bodies of early 3rd instar larvae were dissected for immuno-staining, transcription analysis and protein detection. In larvae over-expressing mutant Rel^K823R^ (*Cg>Rel*^*K823R*^), a strong signal of the HA-tag was detected inside the nuclei of fat bodies (Fig 5A), unlike fat bodies over-expressing Rel^WT^ (*Cg>Rel*^*WT*^), where Relish remained mostly cytoplasmic. When these larvae were systemically infected with *Ecc15* by needle pricking as described [33], Rel^WT^ became nuclear at 6 h as expected, and nuclear localization of mutant Rel^K823R^ increased even further. Quantification indicated that the signal of the HA-tag was significantly higher in the nuclei of fat bodies in SUMOylation-deficient mutant Rel^K823R^ compared to those of Rel^WT^ flies (Fig 5B). The above fat bodies were also dissected for use in a Western blot assay. Over-expression of Rel^WT^ in fat bodies produced a small amount of activated Rel-68 (Fig 5C), which is consistent with previous work [18,50]. However, when mutant Rel^K823R^ was over-expressed, the amount of activated Rel-68 increased (Fig 5C). Quantification indicated that the amount of Rel-68 in the fat bodies of *Cg>Rel*^*K823R*^ was significantly higher than in fat bodies of *Cg>Rel*^*WT*^ flies, even without any immune challenge (Fig 5D). Upon *Ecc15* systemic infection, the amount of Rel-68 produced in the fat bodies of *Cg>Rel*^*K823R*^ was also greater than that of *Cg>Rel*^*WT*^ based on the Western blot assay (Fig 5C) and quantification (Fig 5D). When antibody against Relish [41,42] was used for immuno-staining samples as shown in Fig 5A, Relish signal was detected in nuclei of fat body cells from larvae that received an *Ecc15* injection (S12A Fig). In the fat body cells of *Cg>Rel*^*K823R*^ without *Ecc15* injected, native Relish was not detected in nuclei, indicating that it can not be auto-cleaved like the SUMOylation-deficient mutant Rel^K823R^ even in the same cells (S12B Fig). In summary, loss of SUMOylation seems to enhance Relish cleavage to produce Rel-68 even without immune challenge.

Smt3 with a conserved Gly-Gly motif at the carboxyl end is essential to couple to the target proteins during SUMOylation [9]. *In vitro*, when Rel^WT^ and Rel^K823R^ were over-expressed with or without Smt3-GG in S2 cells, loss of the SUMOylation site in Relish (Rel^K823R^) led to greater amounts of Rel-68 when cells were stimulated with PGN (S13A and S13B Fig). Because Relish transcriptional activity can be assessed using reporter assays in which the Renilla luciferase gene is placed under the control of the Cecropin promoter, this reporter system was co-expressed/transfected with Rel^WT^ (GFP as control) with or without Smt3-GG and Ubc9. When Relish was SUMOylated, luciferase activity was significantly lower than in the un-SUMOylated group upon stimulation with PGN (S14 Fig). This indicates that transcriptional activity of Rel-68 is decreased if Relish is SUMOylated before immune challenge.

We next determined whether production of AMPs was increased after over-expression of Rel^K823R^ in the fat body. Over-expression of Rel^WT^ produced significantly higher levels of AMPs than in control larvae, as reported previously [50]. Over-expression of Rel^K823R^ induced significantly higher transcription of *Att* and *Dpt* in the fat bodies compared with over-expression of Rel^WT^ (Fig 5E). The IMD pathway reporter line *Dpt-lacZ* was used to monitor the impact of over-expression of either Rel^WT^ or Rel^K823R^ in fat bodies of early 3rd instar following immune challenge by *Ecc15* or injury. β-galactosidase activity in fat bodies was assayed to measure the production of Dpt [33]. In unchallenged larvae, over-expression of Rel^K823R^ induced significantly higher β-galactosidase activity than that of Rel^WT^ (Fig 5F). Similarly, over-expression of mutant Rel induced higher reporter activity than wild-type Rel upon *Ecc15* challenge at 6 h. There was no significant difference between reporter activity in injured Rel^WT^ versus Rel^K823R^ flies. Taken together, these data suggest that loss of the SUMOylation site in Relish (Rel^K823R^) leads to significantly higher AMP transcription regardless of immune challenge status. To assess whether this had physiological consequences, we infected larvae with the pathogenic bacteria *Pseudomonas entomophila* by needle pricking and monitored survival. Larvae over-expressing Rel^K823R^ had a higher percentage of survival than those over-expressing Rel^WT^ (Fig 5G). All these data demonstrate that flies lacking the main SUMOylation amino acid in Relish constitutively produce higher levels of Rel-68, which enhance the production of AMPs even in the absence of immune challenge.

### H2Av regulates SUMOylation of Relish by physically interacting with Su(var)2-10

**Fig 6:**
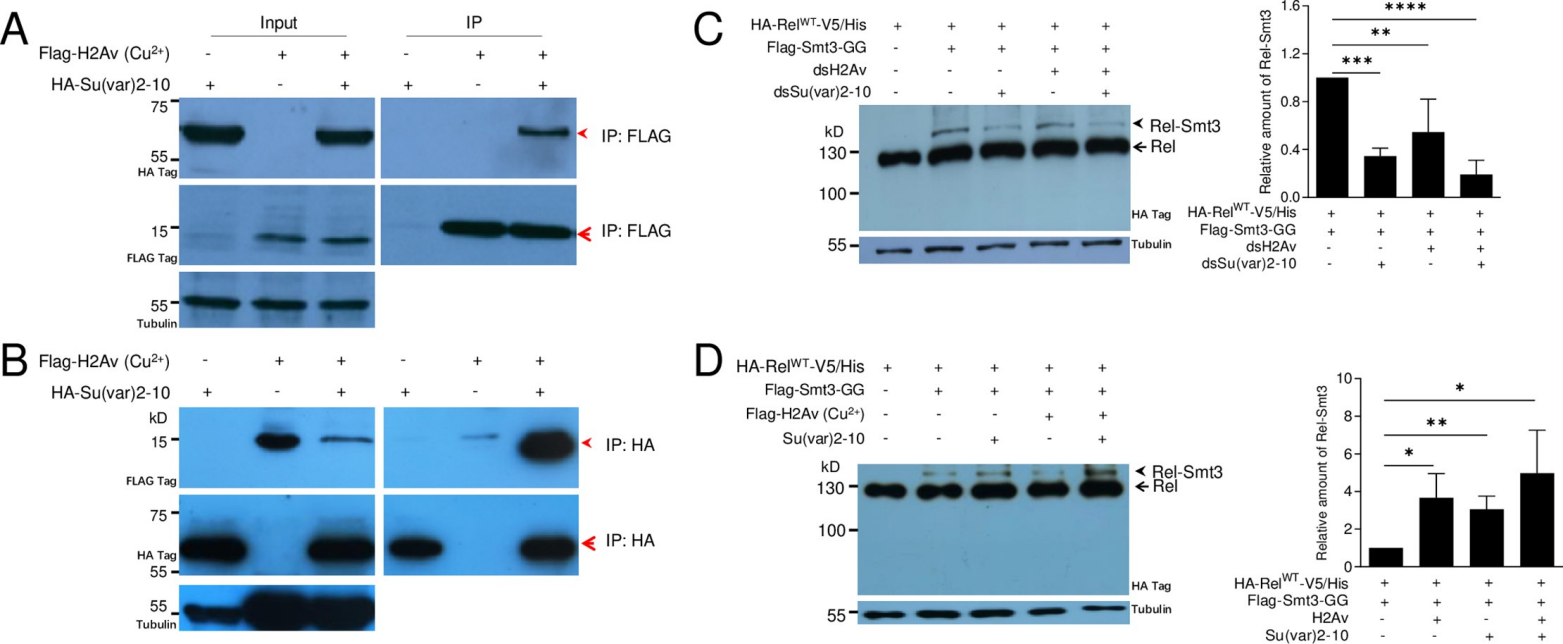
H2Av regulates Relish SUMOylation by interacting with Su(var)2-10. (A, B) Physical interaction between H2Av and Su(var)2-10. Flag-H2Av and/or HA-Su(var)2-10 were expressed in S2 cells using the Cu^2+^-inducible pMT/Bip/V5-His vector. Either antibody against Flag-H2Av (A) or against HA-Su(var)2-10 (B) was used for immunoprecipitation. (C) Knockdown of H2Av and Su(var)2-10 separately or together decreases the amount of SUMOylated Relish (Rel-Smt3). Western blot (Left) shows the change of SUMOylated Relish (Rel-Smt3) after RNAi. Rel-Smt3 was quantified (Right). Rel-Smt3 produced when Rel^WT^ and Smt3 were co-expressed is equal to 1. Data represent the average of at least three independent assays (mean ± SE). (D) Over-expression of H2Av and Su(var)2-10 separately or together significantly increases the amount of SUMOylated Relish (Rel-Smt3). Western blot (Left) shows the change of SUMOylated Relish (Rel-Smt3) after over-expressing H2Av and/or Su(var)2-10. Rel-Smt3 was quantified (Right). Rel-Smt3 produced when Rel^WT^ and Smt3 were co-expressed is equal to 1. Data represent the average of at least three independent assays (mean ± SE). One way ANOVA with Tukey’s multiple comparisons test was performed. *p < 0.05, **p < 0.01, ***p < 0.001, and ****p < 0.0001.

In order to understand how H2Av regulates Relish SUMOylation, H2Av was co-expressed with each gene encoding component of the *Drosophila* SUMOylation pathway and tested using a protein-protein interaction assay. We found that H2Av and Su(var)2-10 could interact with each other when either of them was used as the bait protein (Fig 6A and 6B). Although the reason is unknown, to be used as the bait protein, H2Av or Su(var)2-10 needed to be expressed in a different condition (See the Materials and Methods section for detail). To further analyze the impact of Su(var)2-10 on H2Av regulation of Relish SUMOylation, *H2Av* and/or *Su(var)2-10* were knocked down by RNAi in S2 cells co-expressing Rel^WT^ and Smt3-GG. Compared with the group co-expressing Rel^WT^ and Smt3-GG alone, knockdown of *H2Av* and/or *Su(var)2-10* decreased the amount of Rel-Smt3 (Fig 6C). On the contrary, when H2Av and/or Su(var)2-10 were over-expressed in S2 cells co-expressing Rel^WT^ and Smt3-GG, over-expression of H2Av and/or Su(var)2-10 enhanced the amount of Rel-Smt3 (Fig 6D). In this case, the stable cell line of HA-H2Av had to be used for over-expression since there was almost no expression of HA-H2Av at 48 h during transient expression (S15 Fig). These results demonstrate that H2Av interacts with Su(var)2-10, the E3 component of the *Drosophila* SUMOylation pathway, and that both can regulate Relish SUMOylation.

### SUMOylated Relish may be inhibited for activation upon immune challenge

**Fig 7:**
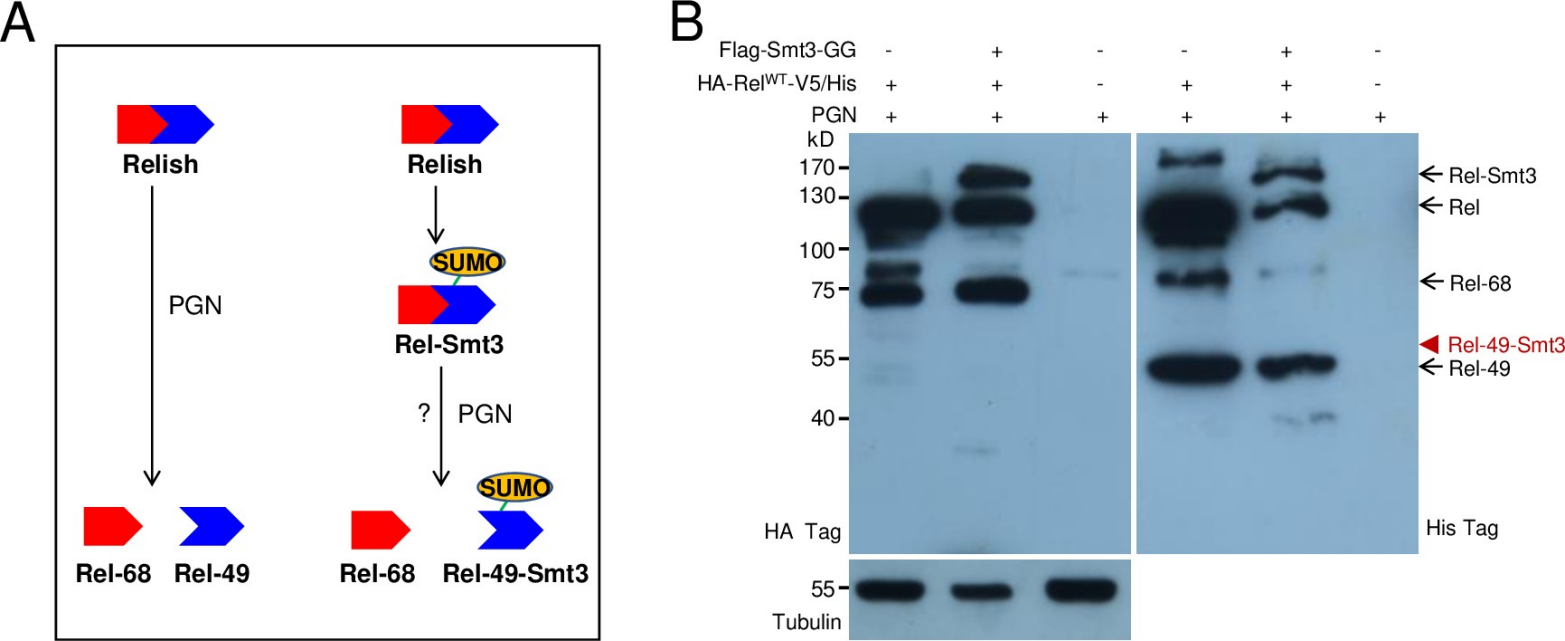
SUMOylated Relish may be inhibited for cleavage. (A) A prediction to show the cleavage of Rel-Smt3 to produce a fragment of Rel-49-Smt3. Wild type Relish can be cleaved into Rel-68 (nuclear translocation) and Rel-49 (in cytoplasm). Upon SUMOylation, Smt3 is mainly covalently coupled to K823 near the C-terminus. Thus, if Rel-Smt3 could also be cleaved like Rel^WT^, it should produce two fragments, Rel-68 and Rel-49-Smt3. Due to the coupled Smt3, the molecular weight of Rel-49-Smt3 should be larger than Rel-49. (B) Undetectable Rel-49-Smt3 on Western blot. By increasing the amount of plasmid containing Smt3-GG and extending the time to collect S2 cells, we increased the amount of Rel-Smt3. Upon PGN addition (PGN alone: PGN + S2 cells), Rel-68 and Rel-49 were all detectable. The proposed band position of Rel-49-Smt3 as indicated by the red arrowhead did not exist. This experiment was repeated three times with similar results.

**Fig 8:**
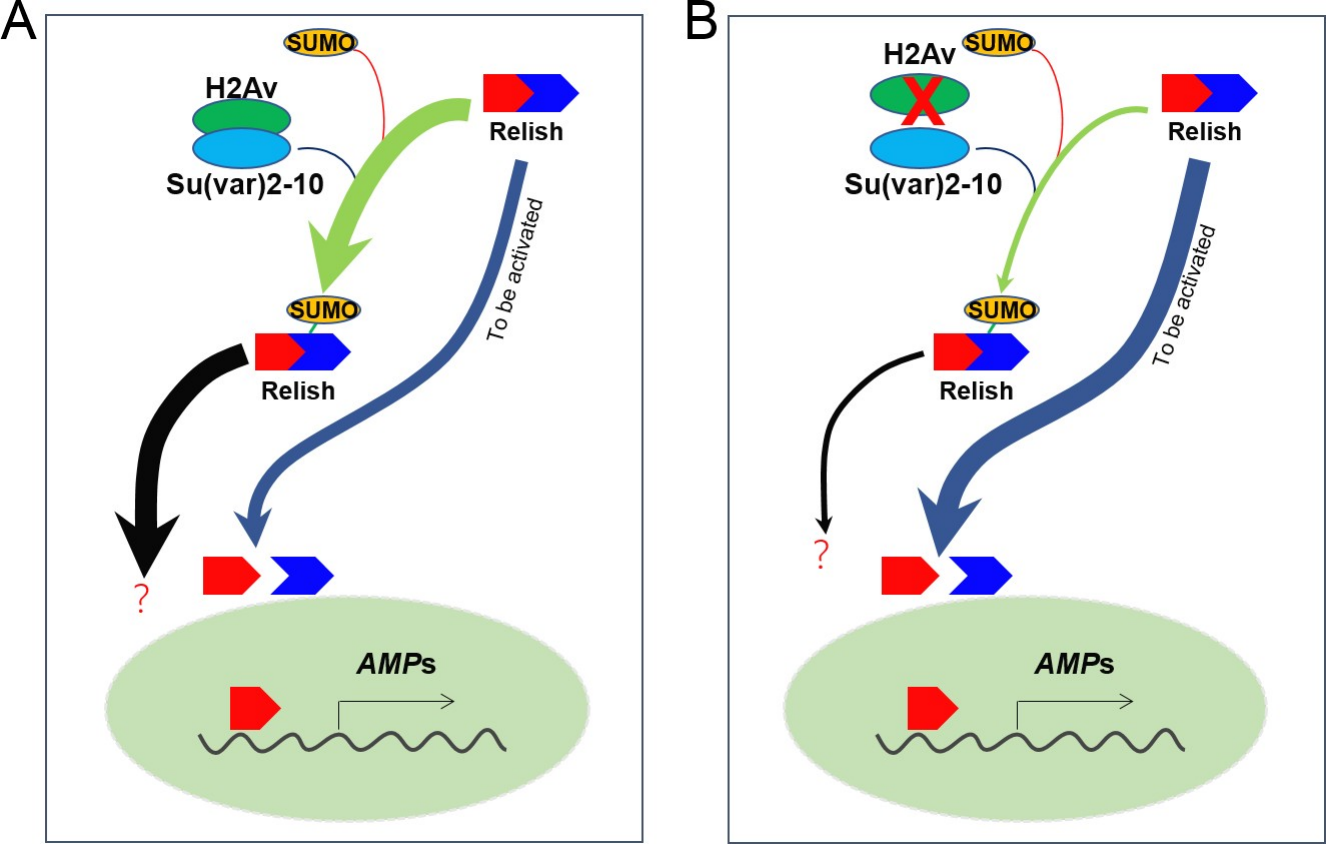
H2Av negatively regulates the IMD pathway to produce AMPs by enhancing Relish SUMOylation. Through interacting with Su(var)2-10, H2Av enhances the SUMOylation of Relish. SUMOylated Relish may not be cleaved upon immune challenge, thus decreasing the amount of cleavable Relish (A). When H2Av loses its function, the amount of SUMOylated Relish decreases correspondingly. Due to this, Relish without modification of SUMOylation is increased above normal levels, which is likely similar to the over-expression of Relish that occurs through genetic methods, and induces its activation by an unknown mechanism (B). Upon immune challenge, Relish that is not SUMOylated can be cleaved and activated by the conventional mechanism. Currently, the fate of SUMOylated Relish is unclear.

After over-expressing HA-Rel^WT^-V5/His in fat bodies (*Cg>Rel*^*WT*^), Rel-Smt3 was pulled down and enriched using HA antibody-conjugated beads. *In vivo*, Rel-Smt3, but not Rel^K823R^-Smt3, could be detected only if large numbers of fat bodies were used as the level was very low (S16A and S16B Fig). The predicted main SUMOylation site (K823R) is located near the C-terminus of Relish. We wondered whether the covalent complex Rel-Smt3 could also be cleaved for activation upon PGN challenge as predicted in Fig 7A. Upon immune challenge, Relish is cleaved at a conserved site to produce two fragments named Rel-68 and *Rel-49* [18,19]. Rel-68 can translocate into nuclei and bind to the promoters of some AMP genes for their transcription [18,19]. Rel-49 remains in the cytoplasm [18,19]. If this were the case, a Rel-49-Smt3 fragment with a higher molecular weight than Rel-49 should be produced at the same time as Rel-68. To test this, a plasmid containing Smt3-GG was co-transferred into S2 cells together with the plasmid containing HA-Rel^WT^-V5/His (Fig 7B). When PGN was added, Rel-68 was produced as detected by the antibody against the HA-tag (HA-tag labels Relish at its N-terminus). When the antibody against the His-tag (His-tag labels Relish at its C-terminus) was used to detect Rel-49, no additional band at a size larger than Rel-49 (the supposed Rel-49-Smt3) was detected (Fig 7B). When antibody against either the HA-tag or His-tag was applied, Rel-Smt3 was detected. Therefore, if Rel-Smt3 were cleaved for activation, it should be technically possible to detect the expected Rel-49-Smt3 band. When the proteasome inhibitor MG132, which cannot prevent the cleavage of Rel, was applied [18], Rel-49-Smt3 was not detected. We conclude that after SUMOylation, Rel-Smt3 is probably inhibited for activation. In summary, we propose that loss of H2Av decreases Relish SUMOylation and consequently increases the amount of Relish without SUMO-modification, allowing Relish auto-activation and transcription of AMPs (Fig 8B). In normal larvae, H2Av-aided SUMOylation of Relish prevents this auto-activation (Fig 8A).

## Discussion

The NF-κB-like transcription factor Relish mediates AMP production downstream of the IMD pathway. Once the IMD pathway is triggered by invading Gram-negative bacteria, a cascade of events leads to cleavage of Relish into two fragments: Rel-68 and Rel-49 [18,19]. Rel-68 contains the Rel homology domain and translocates to the nuclei of fat bodies, hemocytes and midgut cells to induce AMP gene transcription [18]. Rel-49, which contains the inhibitory ankyrin domains, remains in the cytoplasm [18]. The process of IMD activation is fine-tuned by many positive and negative regulators [7,8]. Before cleavage by the caspase Dredd, Relish is phosphorylated by the IKK complex composed of IKKβ and IKKγ (Kenny) [19]. Phosphorylation of Relish is not required for Relish cleavage, nuclear translocation, or DNA binding [19], but it is important for recruiting the RNA polymerase II and for AMP production.

In this work, we show that Relish can be SUMOylated (Fig 4A). Based on bioinformatics predictions and mutational analysis in S2 cells, we identified a main SUMOylation site, K823, in Relish. SUMOylation is abolished in Relish mutant Rel^K823A^ (Fig 4B). Of note, our experimentally verified site K823 is different from the previously predicted site K313, which was based on sequence alignments of Relish with the NF-κB-like transcription factor Dorsal [12]. IKKβ was the first gene of the IMD pathway that was identified as a SUMOylation target [20]. Mutation of the identified site K152 partially decreased SUMOylation on IKKβ and diminished IMD pathway activity. In our study, over-expression of the SUMOylation-deficient Rel^K823A^ *in vivo* led to Relish cleavage, nuclear translocation of Rel-68 and promoted the induction of AMP production, even without bacterial challenge (Fig 5). Over-expression of wild type Relish (Rel^WT^) is known to spontaneously induce some cleavage of Relish [18]. However, over-expression of the SUMOylation-deficient Rel^K823A^ induced levels of AMP much higher than that observed with wild-type Relish (Fig 5D). This result suggests that Relish SUMOylation likely prevents Relish auto-activation. In *Drosophila*, Caspar blocks the Dredd-dependent proteolytic cleavage of Relish and suppresses the nuclear translocation of Relish [51]. In a *casp*^*P1*^ mutant, nuclear Relish was detected even without infection [51], showing that Relish autoactivation is possible. Future work should characterize the role of SUMOylated Caspar and its impact on the IMD pathway. A persistently active immune system is not advantageous to insect development [52,53]. Our results show that after SUMOylation, Rel-Smt3 cannot be cleaved even upon PGN induction of the IMD pathway (Fig 7). Therefore, SUMOylation seems to prevent Relish auto-activation.

The SCF^SKpA/dCull/Slimb^ complex, which acts as a ubiquitin ligase, can promote the degradation of full-length Relish and its cleaved fragments to repress the IMD pathway [50]. SUMO-targeted ubiquitin ligases (STUbL) can detect SUMOylated proteins and modify them by Ubiquitylation, thus linking the SUMOylation and Ubiquitylation pathways [54]. Dgrn (Degringolade) is a protein connecting the ubiquitin and SUMO pathways [15] and *Drosophila* adults lacking Dgrn fail to express AMP genes in response to infection [15]. At this stage, we do not know the fate of SUMOylated Relish. It is unclear if it will be cleaved by Ulp1 to remove the Smt3, or if it will be degraded. Future research is needed to better understand the relationship between Relish SUMOylation and Ubiquitin.

In *Drosophila*, the H2A variant (H2Av) is a homologue of mammalian H2AZ and H2AX [24]. H2A is subjected to different post-transcriptional modifications such as phosphorylation, acetylation, methylation, ubiquitination and SUMOylation [24,45]. H2A is phosphorylated in response to DNA double strand breaks, and then the modified H2A contributes to the repair of DNA damage [23]. In mammals, H2AZ is also involved in chromosome reconstruction and transcriptional regulation. In this work, we demonstrate that loss of function of H2Av promotes the expression of IMD- and Toll-regulated AMP genes (Figs 1 and S6A). We also found that most of the gene encoding components of the Toll-pathway were up-regulated in H2Av (S6C Fig). In addition, Dorsal was detected entering the nuclei of fat body cells (S6D Fig). However, the mechanism regulating the Toll pathway by H2Av needs further study. In *H2Av*^*810*^ larvae, the expression level of the Relish transcript was up-regulated (S7A and S7B Fig) but the other genes of the IMD pathway were not. The active fragment of Rel-68 was detected in fat bodies in *H2Av*^*810*^ larvae (S7C Fig). The commercial antibody against *Drosophila* Relish applied for immuno-staining [41,42] can detect Relish signal in nuclei of fat bodies of *w*^*1118*^ but not *Rel*^*E20*^ mutant larvae after *Ecc15* challenge (S7D Fig). Correspondingly, a Relish signal was also observed in nuclei of fat bodies in *H2Av*^*810*^ larvae using this antibody (S7E–S7G Fig). These results demonstrate that H2Av likely regulates the production of IMD-dependent AMPs through Relish revealing a novel level of fine tuning of the IMD pathway.

Mutants of some SUMOylation-pathway genes (such as Ubc9) exhibit melanotic tumors near the posterior end of larvae [47]. Similarly, *H2Av*^*810*^ also exhibit melanotic tumors in the posterior end of larvae (Fig 1A). We also found that lamellocyte differentiation occurred very similarly in *H2Av*^*810*^ (S1A Fig) and Ubc9 mutants [47]. We then questioned whether H2Av regulates the production of IMD-dependent AMPs via SUMOylation directly or indirectly. Eventually we found that H2Av can regulate Relish SUMOylation after interacting with Su(var)2-10 (Fig 6A and 6B), the E3 of the *Drosophila* SUMOylation pathway. SUMOylated Relish may not be cleaved even after immune challenge (Fig 7). After over-expression of Rel^K823R^ (loss of the main site for SUMOylation) in fat bodies, Rel^K823R^ was cleaved and Relish signal was detected in nuclei *in vivo* (Fig 5A and 5C). For *H2Av*^*810*^, transcription of SUMOylation pathway genes was down-regulated (Fig 3), and SUMOylation activity was thus decreased (S8 Fig). Correspondingly, non-SUMOylated Relish may be accumulated and then cleaved *in vivo*, likely as the observation of Relish over-expression and auto-activation *in vitro* [18]. Therefore, H2Av is an important inhibitor of the production of IMD-dependent AMPs via the involvement of the SUMOylation pathway, which results in lower IMD activity when insects receive no immune challenge. Collectively, the current study identified a new level of regulation in the IMD pathway.

## Materials and methods

### Fly stocks

The fly lines used in this study were: *w*^*1118*^, *Act5C*^*ts*^ (*Act-Gal4; tub-Gal80*^*ts*^, from Zongzhao Zhai), w^*^; P{His2Av-mRFP1}III.*1*, *UAS-H2Av RNAi* (v12768: *w*^*1118*^*; P{GD4747}v12768*, v110598: *P{KK108652}VIE-260B*, BL28966: *y*^*1*^ *v*^*1*^*; P{TRiP*.*HM05177}attP2*), w*; His2Av^*810*^*/TM3*, *Sb*^*1*^ (BL9264), w*; P{His2Av-mRFP1}II.*2* (BL23651; Expression of RFP-tagged His2Av in all cells under the control of His2Av [35]), *CecA1-GFP* (from Bruno Lemaitre), *Rel*^*E20*^ (from Bruno Lemaitre), *Key*^*c02831*^ (BL11044: *w*^*1118*^*; PBac{PB}key*^*c02831*^), *imd*^*1*^, *Dredd*^*B118*^ (*yw; Dredd*^*B118*^, from Bruno Lemaitre), *Cg-gal4* (from Lei Xue), *Dpt-lacZ* (*yw (P(w-*, *Drom-gfP)D4*, *P(ry+*, *Dipt-lacZ)(162*:*7*:*)2*, from Bruno Lemaitre), *Dif*^*1*^ (*y w DD1; Dif*^*1*^), *Tubulin-gal4* (*w*^*1118*^*; tub-Gal4/TM3 Sb*, from Lei Xue), BL9324: y1 w*; P{UAS-lwr.*A}2*, v33685: *w*^*1118*^*; P{GD10017}v33685* and *Actin-gal4* (*w*^*1118*^*; act-Gal4/Cyo*, from Lei Xue). *Drosophila* stocks and experimental crosses were maintained at room temperature on a diet consisting of 0.5% agar, 5% cornmeal, and 5% dry yeast, which was supplemented with 2.857% Nipagin.

### Antibodies and other reagents

The following antibodies and reagents were used in this study: mouse anti-Flag M2 (1:5000, Sigma), rabbit anti-V5 (1:5000, Sigma), mouse anti-His (1:4000, Beyotime), mouse anti-HA (1:5000, Sigma), mouse monoclonal anti-dorsal (AB_528204, Developmental Studies Hybridoma Bank), mouse anti-Tubulin (1:6000, Vazyme), anti-FLAG M2 agarose beads (Sigma), mouse anti-Lamin (ADL67.10), anti-V5 agarose beads (Sigma), anti-HA-agarose beads (Sigma), goat anti-rabbit IgG-HRP (1:10000, Sigma), and goat anti-mouse IgG-HRP (1:10000, Sigma). A commercial antibody against *Drosophila* Relish (Abin1111036, RayBiotech 130–10080) was applied for immuno-staining [41,42] and a polyclonal antiserum against *Drosophila* Relish [40] was applied to a Western blot to detect Relish in *w*^*1118*^ and *His2Av*^*810*^ mutants.

### RNAi in S2 cells

S2 cells were maintained in Schneider’s insect medium (S9895, Sigma) containing 10% fetal bovine serum (GIBCO) at 28°C. RNAi in S2 cells was performed following a published protocol with some changes [55]. Briefly, S2 cells were plated at a density of 1×10^6^ cells in 0.5 ml cell medium. The cultured cell medium was replaced with the same volume of fresh cell medium without FBS 24 h later. Then the cells were transfected with 4 μg dsRNA by using a calcium phosphate transfection method every 24 h for two times. dsRNA corresponding to a fragment of the green fluorescent protein (GFP) was used as a negative control. At 72 h after the first dsRNA transfection, 6 μg of PGN (*E*. *coli* 0111:B4, InvivoGen) was added to each well to induce S2 cell immune responses [19]. At each scheduled time point, S2 cells were collected for qPCR analysis as described below in detail. The primers were listed in S1 Table. dsRNA was synthesized using a T7 RiboMAX™ Express Large Scale RNA Production System (Promega). See S1 Table for the information on the primers.

### Transient co-transfection assay

A transient transfection assay was performed using Effectene (Qiagen) according to the manufacturer’s instructions with some modifications. The final plasmid DNA concentration of each gene was 0.4 μg/ml. S2 cells were collected for a Western blot assay 48 h after transient transfection. Different genes were co-expressed as needed. For the knockdown genes prior to transient transfection, 24 h was permitted after the addition of dsRNA. To activate Relish for cleavage, approximately 6 μg/ml of PGN was added and cells were collected within 2 h. All plasmids for transient transfection are listed in S1 Table.

### Co-immunoprecipitation assay

According to the transient expression assay, there was almost no recombinant Flag-H2Av expressed at 48 h, and it appears to need more time to achieve the same level of recombinant HA-Su(Var)2-10 expressed at 48 h (See S15A and S15B Fig). Although the expression levels were different, both did not interfere with each other when co-transfected into S2 cells at the same time (S15C Fig). After transient co-expression, as the bait protein, HA-Su(Var)2-10 could be used to pull down Flag-H2Av. Although the reason is unknown, for use as the bait protein, much more Flag-H2Av was necessary to pull down HA-Su(Var)2-10 based on our preliminary experiments. Thus, a stable cell line to express Flag-H2Av was constructed as described [56]. Briefly, the FLAG-tagged H2Av construct was cloned into the pMT/V5-His vector, containing the copper-inducible MT promoter. The plasmid and pCoHygro then were co-transfected into S2 cells using Effectene according to the manufacturer’s protocol. At 24 h, the original culture medium was replaced with fresh selection medium containing 10% FBS and 1% hygromycin, followed by a change of medium one time 2–3 days later to create a stable S2 cell line to express H2Av-Flag.

The HA-Su(Var)2-10 construct, cloned into the pAC5.1B vector, was transfected into the above stable cell line expressing FLAG-tagged H2Av and into the parental S2 cells as described above in detail. At the same time, all cells were stimulated with 100 μM Copper sulfate for 48 h. The cells were lysed in lysis buffer (20 mM Tris at pH 7.6, 150 mM NaCl, 2 mM EDTA, 10% Glycerol, 1% Triton X-100, 1 mM DTT, NaVO4, glycerol 2-phosphate and protease inhibitors). Cell lysates were immunoprecipitated with anti-FLAG antibody (Flag-H2Av as the bait protein) at 4°C overnight followed by incubation with protein G-Sepharose beads (GE Healthcare) at 4°C for 1 h. These beads were washed three times with the lysis buffer. For immunoblot analysis, anti-FLAG and anti-HA antibodies were used.

To use HA-Su(Var)2-10 as the bait protein, the same amount of plasmids containing Flag-H2Av and HA-Su(Var)2-10 were transiently co-transfected into S2 cells with 100 μM Copper sulfate added. Proteins were expressed for 48 h and harvested for pull-down as described above. Anti-FLAG and anti-HA antibodies were used for immunoblot analysis.

### RNAi analysis *in vivo*

RNAi knockdown experiments were performed by crossing flies with the His2Av or Ubc9 RNAi hairpin under the UAS regulatory sequence or *w*^*1118*^ to *Act5C*^*ts*^ or *act-gal4* or *tub-gal4*. The progeny were raised at 18°C until eclosion and fed for 3 days. The adults were then transferred and held at 29°C for 3 days. For RNAi knockdown in the larvae, the adults were permitted to lay eggs at 18°C. After hatching, the progeny were transferred and held at 29°C. RNAi knockdown driven by act-gal4 or tub-gal4 was to observe the melanotic tumors in larvae. RNAi knockdown driven by *Act5C*^*ts*^ was used for qPCR to analyze gene change in fat bodies. See S1 Table for the information on primers.

### Germ-free larvae

Germ-free animals were prepared as described [57]. Briefly, dechorionated embryos were sterilized three times for 1 min using Disinfectant Liquid (1:30; Walch) each time, and sterilized again for 1 min using 2–2.5% sodium hypochlorite (Sigma), followed by washing twice in 70% ethanol for 1 min each time and twice in 0.1% PBST (0.1% TritonX-100 in PBS). The larvae then fed on axenic foods prepared as described [58] and routinely checked for bacterial contamination by plating the lysates obtained from the individuals onto LB media and incubating at 37°C overnight to ensure that they were germ-free. In the meantime, PCR analysis on fly homogenates was also performed using 16S rRNA primers. Fat bodies were dissected before the wandering stage for qPCR analysis.

### Quantitative measurements of β-galactosidase activity

The method as described by Neyen et al was followed with some modifications [33]. Fat bodies from 15 female adults were dissected and homogenized in 750 μl lysis buffer (Beyotime) and centrifuged for 10 min at 10,000 rpm at 4°C. The protein concentration of the supernatant was determined by a Bradford assay [59]. The supernatant (30 μl) was mixed with 200 μl ortho-Nitrophenyl-b-galactoside (ONPG, 0.35 mg/ml) solution prepared in Z buffer (60 mM Na2HPO4, 60 mM NaH2PO4, 10 mM KCl, 1 mM MgSO4, 50 mM β-mercaptoethanol, pH = 8.0) on ice. The 96-well plates were then incubated in a heated microplate reader (37°C) and measurements taken every 5–10 min at 420 nm. β-galactosidase activity was calculated as described [33].

### Nuclear and cytoplasmic protein extraction

The Nuclear and Cytoplasmic Protein Extraction Kit (P0027, Beyotime) was used for separating and enriching nuclear and cytoplasmic proteins of the parental S2 cells and the S2 stable cell line that expressed Flag-H2Av respectively. The methods described by the manufacturer were followed. Briefly, S2 cells were suspended and separated for 5 min at 5000 rpm at room temperature. All culture medium was discarded. Approximately 200 μl Buffer A (containing 1 mM PMSF) was applied to suspend the cells precipitated from 20 μl cells by vortexing for 5 seconds. After incubation on ice for 10–15 min, 10 μl of Buffer B was added, followed by vortexing for 5 seconds, incubating on ice for 1 min and then vortexing again for another 5 seconds. The lysate was centrifuged at 12000 g for 5 min at 4°C. The supernatant contained the cytoplasmic proteins. The cell debris was then suspended in 50 μl Buffer B containing 1 mM PMSF, vortexed for 15–30 seconds and incubated on ice for 10 min. This lysate was centrifuged at 12000 g for 10 min at 4°C and the resulting supernatant contained nuclear proteins. The protein concentration was determined by Bradford assay [59] and samples were aliquoted for storage at -80°C. These samples were used for detection of H2Av, Lamin and rH2Av-FLAG by Western blot respectively.

### Antibody preparation

*Drosophila* Smt3 recombinant protein was purified from *E*. *coli* cells. The rats for immunization were initially screened by Western blot using cell lysates from different tissues of *Drosophila* larvae and adults including the purified recombinant Smt3. The rats with no detectable IgG to *Drosophila* proteins and recombinant Smt3 were selected for the subsequent immunization. The anti-Smt3 antiserum was collected and the aliquot was stored at -80°C.

### Luciferase-reporter assay

Based on a published paper [60], the Cecropin promoter sequence (from -760 to +62) was cloned to make the vector pGL3-CecA1-Promoter. The following plasmids and amounts used for this experiment were as follows: pGL3-CecA1-Promoter (0.3 μg), pRL (0.02 μg) as an internal standard, pAC5.1B-HA-Rel^WT^-V5/His (0.4 μg), pAC5.1B-Flag-SUMO^GG^ (0.4 μg), and pAC5.1B-Ubc9 (0.4 μg). The above plasmids were transiently transfected into S2 cells as described above. At 36 h, PGN (10 μg/ml) was added and incubated for 1 and 2 h. Cells were collected and divided into two aliquots, one for Western blot assay to detect Relish SUMOylation and another for a luciferase assay. S2 cells were lysed and the luciferase assay was performed using the Dual Luciferase Reporter Assay System (E1910, Promega) according to the manufacturer’s instructions and analyzed in a luminometer.

### Quantitative RT-PCR (qRT-PCR) analysis

Total RNA was extracted from tissues or cells using TRIzol (Invitrogen), then total RNA was quantitated. The cDNA was prepared using the ReverTra Ace qPCR RT Master Mix with gDNA Remover (TOYOBO) and with oligodT and random primers. Hieff qPCR SYBR Green Master Mix (No Rox) and an ABI7500 system (Applied Biosystems) were used for quantitative RT-PCR. Quantification was normalized to endogenous ribosomal protein Rp49 mRNA.

### Vector construction and generation of transgenic lines

The PCR fragments of wild type Relish (*HA-Rel*^*WT*^*-V5/His*) and mutant Relish with the primary SUMOylation site lost (*HA-Relish*^*K823R*^*-V5/His*) were subcloned into the pUAST-attB vector. This construct was injected into y[1] M{vas-int.Dm}ZH-2A w[*]; P{CaryP}attP40 (Core Facility of *Drosophila* Resource and Technology, SIBCB, CAS). The adults with red eyes were crossed with *Sp/CyO*,*Kr-GFP;MKRS/TM6B* to obtain lines to over express *HA-Rel*^*WT*^*-V5/His* (*UAS-HA-Rel*^*WT*^*-V5/His/CyO*,*Kr-GFP;MKRS/TM6B*) and *HA-Relish*^*K823R*^*-V5/His* (*UAS-HA-Relish*^*K823R*^*-V5/His/CyO*,*Kr-GFP;MKRS/TM6B*) respectively.

### SUMOylation amino acid prediction and mutation

All the potential SUMOylated amino acids were predicted using the SUMOsp online tool (http://sumosp.biocuckoo.org). Using the pAc5.1B-HA-Relish-V5/His vector as the template, the point mutation of lysine (K) to arginine (R) was PCR-generated by using site-directed mutagenesis (QuikChange, Stratagene). The corresponding vector was used for over-expression in S2 cells, or sub-cloned for *Drosophila* transgenic line generation.

### Fat body preparation for immunoblotting and immunoprecipitation

Fat bodies from 30 larvae (early 3rd instar before the wandering stage) were dissected in sterile 0.85% NaCl solution. Approximately 150 μl RIPA Lysis Buffer (P0013B, Beyotime) containing 1 mM PMSF was added for lysis on ice. The cell lysate was centrifuged at 13000 g for 3 min at 4°C. The supernatant under the fat body oil was carefully removed for immunoblotting and immunoprecipitation.

Fat bodies from larvae (*Cg>HA-Rel*^*WT*^*-V5/His*) were lysed as above. The protein concentration was determined by the Bradford assay [59]. In order to pull down SUMOylated Relish, 5 μg antibodies against the HA-tag were incubated with 25 μl Protein A/G Agarose (Beyotime) followed by incubation with 500 μg total protein for 6 h at 4°C. The above beads were washed 5 times. The beads were suspended in 1×SDS loading buffer and boiled, and the supernatant was used for immunoblotting.

### Infection experiments

Bacterial infection was carried out by pricking second or third-instar (before the wandering stage) larvae or 3-day-old adults, which were pre-paralyzed on a clean paper on ice, with a thin needle dipped into a concentrated solution of the following bacterial strains: *Erwinia carotovora carotovora 15* (*Ecc15*) for GFP fluorescence observation and quantification in whole larvae and fat bodies (*Cec-GFP and Cec-GFP;H2Av*^*810*^) and *Pseudomonas entomophila* for survival analysis in adults (*Cg>HA-Rel*^*WT*^*-V5/His and UAS-HA-Relish*^*K823R*^*-V5/His*). After removal from ice for recovery, the dead larvae and adults were discarded.

At 6 h after infection with *Ecc15*, the whole larvae were observed using fluorescent stereomicroscopy (Olympus BX51) for the same exposure time. Fat bodies were also removed and fixed in 4% paraformaldehyde solution. The nuclei were counter-stained with 4,6-diamidino-2-phenylindole (DAPI). GFP fluorescence was observed and imaged using fluorescent microscopy with the corresponding filters (Olympus BX51, Japan) for the same exposure time. GFP fluorescence of whole larvae or fat bodies was quantified using ImageJ software.

Survival experiments were performed with 30 adults at 25°C for each fly line that was maintained using the same conditions. Surviving flies were transferred to fresh vials and counted immediately when any adult died after *P*. *entomophila* infection. Eventually the number of died adults within each 10 h was applied for statistics analysis later.

### Immuno-staining and confocal microscopy

Germ-free larvae (*Cg>HA-Rel*^*WT*^*-V5/His* and *UAS-HA-Relish*^*K823R*^*-V5/His*) at the 3rd instar were immune challenged with *Ecc15* and dissected at 6 h post infection. Fat bodies from those larvae were dissected and fixed in 4% paraformaldehyde for 2 h. Fat bodies were then incubated in solution with 0.1% Triton X-100 prepared in 0.1% citrate sodium for 30–60 min. Fat bodies were incubated with the 1st antibody against the HA-tag (1:500) at 4°C overnight followed by incubation with the Goat-anti-Rabbit 2nd antibody Alexa Fluor 594 (1:500) for 2 h at room temperature. DAPI (5 μg/ml) was used to stain the nuclei. The above antibodies were prepared in PBTA buffer (0.1% Tween 20 and 1% BSA in PBS buffer). Washes between antibodies were done in PBTA buffer for three times with each for 5–10 minutes. The fat bodies were mounted for imaging using an Olympus FV1000. The fluorescence level of nuclear translocated Rel-68 was measured within the nucleus whose boundary was defined from the DAPI image. To detect Lamin in nuclei, adult midguts were dissected and immuno-stained using the primary antibody against Lamin.

### Western blotting, quantification and statistical analyses

Samples were subjected to 12% SDS-PAGE and transferred to a polyvinylidene difluoride membrane. The proteins were detected using HRP-linked anti-mouse IgG (GE Amersham) or anti-rabbit IgG (Bio-Rad) and the ECL detection system (Thermo Scientific). The intensity of protein bands was measured using ImageJ software and the relative amount was calculated.

### Statistical analysis

In this study, one way ANOVA with Tukey’s multiple comparisons test or two-tailed Student’s t-test was performed. Log-rank test was used for survival statistical analysis. All analyses were performed with GraphPad Prism. Differences were considered significant if p values were less than 0.05 (*), 0.01 (**), 0.001 (***), or 0.0001 (****).

## Supporting information

S1 Fig Loss of H2Av induces lamellocyte differentiation and expression of PPO3. (A) Lamellocytes in *H2Av*^*810*^ mutant larvae. Hemocytes were released from *w*^*1118*^ and *H2Av*^*810*^ mutant into PBS buffer respectively. Many lamellocytes (arrow-indicated) appeared in *H2Av*^*810*^ mutant larvae. (B) The phenotype of lamellocyte differentiation is partially rescued by crossing *H2Av-mRFP* and *H2Av*^*810*^. Very few lamellocytes were detected in *w*^*1118*^ and *H2Av-mRFP* larvae. The percentage of lamellocytes in *H2Av*^*810*^ mutant is significantly higher than in *w*^*1118*^. When *H2Av-mRFP* was crossed with *H2Av*^*810*^, lamellocyte differentiation was significantly but not totally inhibited. Therefore, expression of *H2Av-mRFP* partially rescued the phenotype of lamellocyte differentiation following the loss of H2Av. Data represent the average of at least three independent assays (mean ± SE). (C) Transcription of PPO3 in *H2Av*^*810*^ mutant larvae. cDNA of hemocytes from *w*^*1118*^ and *H2Av*^*810*^ mutant larvae, which were *Ecc15* immune-challenged or not, were used as templates for PCR analysis. PPO3 transcription was observed in *H2Av*^*810*^ mutants with (+) or without (-) immune-challenge. One way ANOVA with Tukey’s multiple comparisons test was performed. ***p < 0.001 and ****p < 0.0001. Bar: 20 μm.
(TIF)

S2 Fig **qPCR analysis of *ecdysone 20-monooxygenase* (A) and *akirin* (B) in whole bodies of *w***^***1118***^**and *H2Av***^***810***^**second-instar larvae.***Ecdysone 20-monooxygenase* encodes the terminal gene to produce 20E in the pathway of ecdysteroid production [29,30]. *akirin* is a positive regulator of the IMD pathway by increasing activity of Rel in the nucleus [43,44].Two-tailed Student’s t-test was performed. Data represent the average of at least three independent assays (mean ± SE). **p < 0.01 and ****p < 0.0001.
(TIF)

S3 Fig Knock-down of H2Av in S2 cells enhances AMP production after challenge. *H2Av* and *key* (an important component of the IMD pathway) were knocked down for 2 days. After that, PGN (A) was added separately for different periods. S2 cells were then collected for qPCR analysis of *Att*, *Cec* and *Dpt* genes. *Key* knock-down abolished production of AMPs. After knock-down of H2Av, the production of each AMP was significantly higher than the control at each time point after PGN application. (B, C) The efficiency of knockdown of *key* and *H2Av* was assayed respectively before immune challenge. Two-tailed Student’s t-test was performed. Data represent the average of at least three independent assays (mean ± SE). *p < 0.05, **p < 0.01, and ***p < 0.001.
(TIF)

S4 Fig Knock-down of H2Av *in vivo* enhances AMP production in larvae and adults. Three RNAi lines of H2Av were separately driven by *Act5C*^*ts*^ and reared at 18°C. For larvae (A, B), offspring were brought to 29°C one day after hatching and fed for 2–3 d before dissecting fat bodies. For adults (C, D), offspring were brought to 29°C at day 3 after eclosion. Fat bodies were dissected and collected 3 d later. qPCR analysis of different AMPs was performed. The efficiency of knock-down of *H2Av* in larvae (B) and adults (D) was also analyzed. Knockdown of H2Av in either larvae or adults can significantly increase AMP production. One way ANOVA with Tukey’s multiple comparisons test was performed. Data represent the average of at least three independent assays (mean ± SE). *p < 0.05, **p < 0.01, ***p < 0.001, and ****p < 0.0001.
(TIF)

S5 Fig Knockdown of *H2Av* produces melanotic tumors in larvae. Three RNAi lines of H2Av were driven by *Act-gal4* or *Tub-gal4*. Melanotic tumors as arrows indicated were observed near the posterior end of the larvae, a phenotype which is similar to *H2Av*^*810*^/*H2Av*^*810*^ mutant larvae. Knockdown of GFP was the control. Bar: (A-D) and (E-H) 1 mm.
(TIF)

S6 Fig Loss of H2Av upregulates the production of Drosomycin (Drs). (A) Drs was significantly up-regulated in *H2Av*^*810*^ mutant larvae, which can be rescued by expression of H2Av-mRFP. (B) Knock-down of H2Av enhances the production of Drs in larvae. Two RNAi lines of H2Av were driven by Tub-gal4. qPCR analysis shows significant upregulation of Drs in the fat bodies after RNAi. (C) Up-regulation of Toll pathway components in *H2Av*^*810*^ mutant larvae. Fat bodies of *H2Av*^*810*^ mutant and *w*^*1118*^ second-instar larvae were dissected for qPCR analysis. Although the results show that main Toll pathway genes are upregulated, further study is needed to determine the role of the Toll pathway since Drs is synergistically regulated by the IMD and Toll pathways [2,37,38]. Data represent the average of at least three independent assays (mean ± SE) in (A-C). (D) Nuclear translocation of Dorsal in the fat bodies of *H2Av*^*810*^ mutant larvae. Dorsal and Dif are transcription factors of the Toll pathway that will translocate into the nuclei if this pathway is activated. Compared with *w*^*1118*^, Dorsal signal was detected in the nuclei of fat bodies of *H2Av*^*810*^ mutant larvae (arrow-indicated). One way ANOVA with Tukey’s multiple comparisons test (A, B) or two-tailed Student’s t-test (C) was performed. **p < 0.01, ***p < 0.001, and ****p < 0.0001. Bar: 50 μm.
(TIF)

S7 Fig Cleavage of Relish in the *H2Av*^*810*^ mutant. (A, B) qPCR analysis of *Relish* in the fat bodies of larvae conventionally reared (A) or germ-free (B) before the wandering stage. (C) Western blotting shows Relish and Rel-68 in *w*^*1118*^ (received *Ecc15* challenge or not as a positive or negative control) and *H2Av*^*810*^ mutants (without *Ecc15* challenge). Polyclonal antiserum against *Drosophila* Relish [40] was used for the Western blotting assay and active Rel-68 was detected in *H2Av*^*810*^. The arrowhead and arrow point to Relish (Rel) and Rel-68 respectively. (D) Commercial antibody against *Drosophila* Relish (Abin1111036, RayBiotech 130–10080) was applied for immuno-staining fat bodies dissected from *w*^*1118*^ and *Rel*^*E20*^ received immune challenged according to the published papers [41,42]. Using this commercial antibody, we detected Relish signal in nuclei of fat bodies of *w*^*1118*^ but not *Rel*^*E20*^ mutant larvae. (E) Immuno-staining to show the distribution of Relish in fat body cells and Rel-68 in nuclei of larval fat bodies of *H2Av*^*810*^ mutant. The arrows indicate cells with Relish signal inside nuclei. (F-G) Quantification of Relish in whole fat body cells (F) and Rel-68 in nuclei (G). The amount of Rel or Rel-68 produced from fat bodies of *w*^*1118*^ is equal to 1 for quantification. Two-tailed Student’s t-test was performed. Mean values are presented ±SE. **p < 0.01, ***p < 0.001, and ****p < 0.0001. Bar: 20 μm.
(TIF)

S8 Fig Detection and quantification of SUMOylated proteins in *w*^*1118*^ and *H2Av*^*810*^ larvae. Proteins lysed from fat bodies of three larvae were loaded for each lane. The polyclonal antibody against Smt3 used in this study can detect SUMOylated proteins. Each blot was quantified using ImageJ from the NIH (https://imagej.nih.gov/ij/docs/guide/146-30.html). The lanes were plotted as indicated for quantification. The histogram of each lane was also placed under the corresponding blot. The area of each peak was enclosed for calculating the area that was listed below. Each *w*^*1118*^ = 1, and the relative density was calculated. This experiment was repeated 3 times independently.
(TIF)

S9 Fig Knockdown of *Ubc9* induces Relish nuclear translocation and AMP expression. (A) Knockdown of *Ubc9* produces melanotic tumors in larvae. *Ubc9* was knocked down or over-expressed via *Act5C*^*ts*^. Melanotic tumors, as arrows indicate, were observed near the posterior end of *Ubc9* knockdown larvae but not the control and Ubc9 over-expression larvae, a phenotype which is similar to *H2Av*^*810*^*/H2Av*^*810*^ mutant larvae. (B) qPCR and western blot to show the transcription level of Ubc9 and the SUMOylated proteins after knockdown or over-expression of Ubc9. Data represent the average of three independent assays (mean ± SE). (C) Commercial antibody against *Drosophila* Relish (Abin1111036, RayBiotech 130–10080) was applied for the immuno-staining of fat bodies dissected from the above larvae that received no immunochallenge. Relish signal was detected in nuclei of fat bodies of *Ubc9* knockdown larvae but not the control and Ubc9 over-expression larvae. (D) In Ubc9 knockdown larvae but not the control and Ubc9 over-expression larvae the production of AMPs was enhanced. Data represent the average of three independent assays (mean ± SE). One way ANOVA with Tukey’s multiple comparisons test was performed. **p < 0.01 and ***p < 0.001. Bar: (A) 0.5 mm. (B) 50 μm.
(TIF)

S10 Fig H2Av in cytoplasm. (A) Recombinant H2Av-Flag (rH2Av-Flag) was transfected into S2 cells to make a stable cell line using the Cu^2+^ inducible pMT/V5-His vector as described [56]. S2 cells stimulated or not to express rH2Av-Flag were lysed and the cytoplasmic (C) and nuclear (N) fractions were separated. Western blots using antibody against Flag-tag and H2Av. * indicate that one nuclei protein was non-specifically detected by the FLAG antibody. Both endogenous H2Av and newly-expressed rH2Av-Flag were detected in the cytoplasm and nuclei. (B) Detection of rH2Av-Flag in cytoplasm and nuclei. When rH2Av-Flag was induced to express, S2 cells were fixed for immuno-staining and observed using confocal microscopy. The arrows indicate Flag-staining signal in the cytoplasm and nuclei of some S2 cells. The red arrowhead indicates the Flag signal in the nucleus but not in the cytoplasm, which might be due to the low amount of rH2Av-Flag expressed. The white arrowhead indicates one S2 cell without rH2Av-Flag expression due to no Flag signal staining. The immuno-staining also demonstrates that there is cytoplasmic H2Av in *Drosophila* cells. (C) Detection of lamin in the nuclei of adult midguts. Lamin is a nuclei-associated protein and the anti-lamin antibody used for immuno-staining showed its location around the nuclei of midgut cells. (D) Detection of lamin in the cytoplasmic and nuclear fractions. There was no lamin signal in the cytoplasmic fraction as shown in (A), indicating no contamination of nuclear proteins during separation.
(TIF)

S11 Fig Relish SUMOylation and cleavage are independent. To avoid Relish auto-activation [18] which may interfere with the influence of Relish SUMOylation on cleavage, cells were collected after being transiently transfected for 30 h. Relish is cleaved at D545 for activation [19]. When D545 was mutated (Rel^D545A^) and over-expressed in S2 cells, it could not be cleaved following PGN stimulation for 2 h. However, SUMOylation was not affected. K823 is the main site for SUMOylation. When mutant Rel^K823R^ (loss of SUMOylation) was over-expressed in S2 cells, it could be cleaved after PGN stimulation. Double mutant Rel^D545A/K823R^ could neither be SUMOylated nor cleaved. The arrows indicate the positions of Rel-Smt3, Rel (wild type and different mutants) and Rel-68 respectively.
(TIF)

S12 Fig Detection of native Relish in fat bodies with *HA-Rel*^*WT*^*–V5/His* and *HA-Rel*^*K823R*^*-V5/His* over-expressed using the *Cg-gal4* driver. Fat bodies from larvae that received *Ecc15* injection (A) or not (B) were fixed for Relish immuno-staining. For fat bodies from *HA-Rel*^*WT*^*–V5/His* and *HA-Rel*^*K823R*^*-V5/His* over-expressed larvae, if *Ecc15* was not injected, no signal of native Relish was detected inside nuclei using the commercial antibody against Relish [41,42].
(TIF)

S13 Fig Relish SUMOylation does not promote cleavage. *HA-Rel*^*WT*^*-V5/His* and *HA-Rel*^*K823R*^*-V5/His* (loss of main site for SUMOylation) were separately co-expressed with Smt3-GG or not as indicated for 48 h (A), PGN was applied for different periods. Western blot shows the cleavage of Rel^WT^ and Rel^K823R^. The arrows indicate the positions of Rel-Smt3, Rel (Rel^WT^ and Rel^K823R^) and Rel-68 separately. The production of Rel-68 was quantified for each treatment (B). Loss of potential SUMOylation can enhance Relish cleavage. Data represent the average of three independent assays (mean ± SE). Two-tailed Student’s t-test was performed. *p < 0.05.
(TIF)

S14 Fig Relish SUMOylation decreases its transcriptional activity. Reporter assays using the luciferase gene under the control of the Cecropin promoter. GFP or Rel^WT^ alone were over-expressed with the above reporter system. Rel^WT^, Smt3-GG and Ube 9 were co-expressed with the above reporter system for 48 h. Then PGN was applied to induce Relish activation and cleavage. At each indicated time point, significantly higher luciferase activities were detected in the treatment without obvious SUMOylation (Rel^WT^ alone) compared to those with SUMOylation (Rel^WT^, Smt3-GG and Ubc9 co-expressed). Data represent the average of three independent assays (mean ± SE). Two-tailed Student’s t-test was performed. *p < 0.05, **p < 0.01, and ****p < 0.0001.
(TIF)

S15 Fig Transient expression of Flag-H2Av lag behind that of HA-Su(Var)2-10. The sequences of Flag-H2Av and HA-Su(Var)2-10 were inserted into the pMT/V5-His vector respectively for transient expression in S2 cells. The same number of S2 cells were collected and lysed in the same volume of 1X SDS loading buffer at each time point. HA-Su(Var)2-10 was expressed in a large amount at 48 h as detected using a Western blot assay (A). There was almost no expression of Flag-H2Av at 48 h until 60–72 h (B). Transient co-expression of Flag-H2Av and HA-Su(Var)2-10 did interrupt each other (C). Therefore, the expression of Flag-H2Av lags behind that of HA-Su(Var)2-10 for an unknown reason. Thus, a stable cell line to express Flag-H2Av was constructed for *in vitro* experiments. Blank: S2 cells were transiently transfected with the same amount of pMT/V5-His vector without any gene inserted.
(TIF)

S16 Fig Detection of SUMOylated Relish *in vivo*. HA-Rel^WT^-V5/His (A) or HA-Rel^K823R^-V5/His (B) was over-expressed in larval fat bodies using *Cg-gal4*. SUMOylated Relish was enriched using antibody against HA-tag conjugated beads. Fat bodies from 50 larvae were collected and lysed for pull-down and antibody against the HA-tag was used in a Western blot to detect the SUMOylated Relish. The arrows indicate Rel-Smt3 and Rel respectively. *In vivo*, SUMOylated HA-Rel^WT^-V5/His-Smt3 (Rel-Smt3) but not HA-Rel^K823R^-V5/His-Smt3 was detected but the amount was low.
(TIF)

S1 Table Primers for qPCR and dsRNA synthesis. (XLSX)
