# Supplementary figures and images for: Drosophila H2Av negatively regulates the activity of the IMD pathway via facilitating Relish SUMOylation
Source: PLoS Genet. 2021 Aug 9;17(8):e1009718. doi: 10.1371/journal.pgen.1009718 (PMC8376203; doi:10.1371/journal.pgen.1009718)

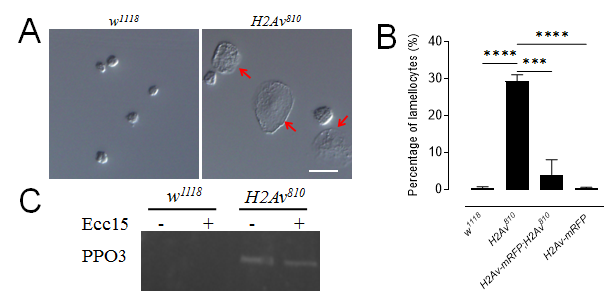

Supplement: S1 Fig — (A) Lamellocytes in H2Av810 mutant larvae. Hemocytes were released from w1118 and H2Av810 mutant into PBS buffer respectively. Many lamellocytes (arrow-indicated) appeared in H2Av810 mutant larvae. (B) The phenotype of lamellocyte differentiation is partially rescued by crossing H2Av-mRFP and H2Av810. Very few lamellocytes were detected in w1118 and H2Av-mRFP larvae. The percentage of lamellocytes in H2Av810 mutant is significantly higher than in w1118. When H2Av-mRFP was crossed with H2Av810, lamellocyte differentiation was significantly but not totally inhibited. Therefore, expression of H2Av-mRFP partially rescued the phenotype of lamellocyte differentiation following the loss of H2Av. Data represent the average of at least three independent assays (mean ± SE). (C) Transcription of PPO3 in H2Av810 mutant larvae. cDNA of hemocytes from w1118 and H2Av810 mutant larvae, which were Ecc15 immune-challenged or not, were used as templates for PCR analysis. PPO3 transcription was observed in H2Av810 mutants with (+) or without (-) immune-challenge. One way ANOVA with Tukey’s multiple comparisons test was performed. ***p < 0.001 and ****p < 0.0001. Bar: 20 μm. (TIF) [file pgen.1009718.s001.tif]

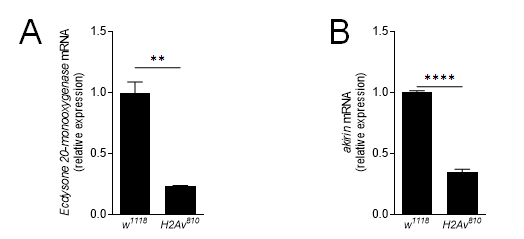

Supplement: S2 Fig — qPCR analysis of ecdysone 20-monooxygenase (A) and akirin (B) in whole bodies of w1118and H2Av810second-instar larvae.Ecdysone 20-monooxygenase encodes the terminal gene to produce 20E in the pathway of ecdysteroid production [29,30]. akirin is a positive regulator of the IMD pathway by increasing activity of Rel in the nucleus [43,44].Two-tailed Student’s t-test was performed. Data represent the average of at least three independent assays (mean ± SE). **p < 0.01 and ****p < 0.0001. (TIF) [file pgen.1009718.s002.tif]

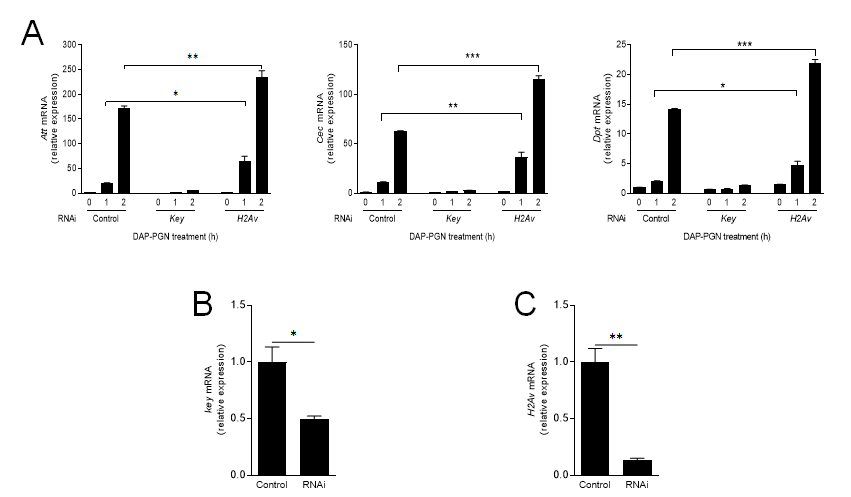

Supplement: S3 Fig — H2Av and key (an important component of the IMD pathway) were knocked down for 2 days. After that, PGN (A) was added separately for different periods. S2 cells were then collected for qPCR analysis of Att, Cec and Dpt genes. Key knock-down abolished production of AMPs. After knock-down of H2Av, the production of each AMP was significantly higher than the control at each time point after PGN application. (B, C) The efficiency of knockdown of key and H2Av was assayed respectively before immune challenge. Two-tailed Student’s t-test was performed. Data represent the average of at least three independent assays (mean ± SE). *p < 0.05, **p < 0.01, and ***p < 0.001. (TIF) [file pgen.1009718.s003.tif]

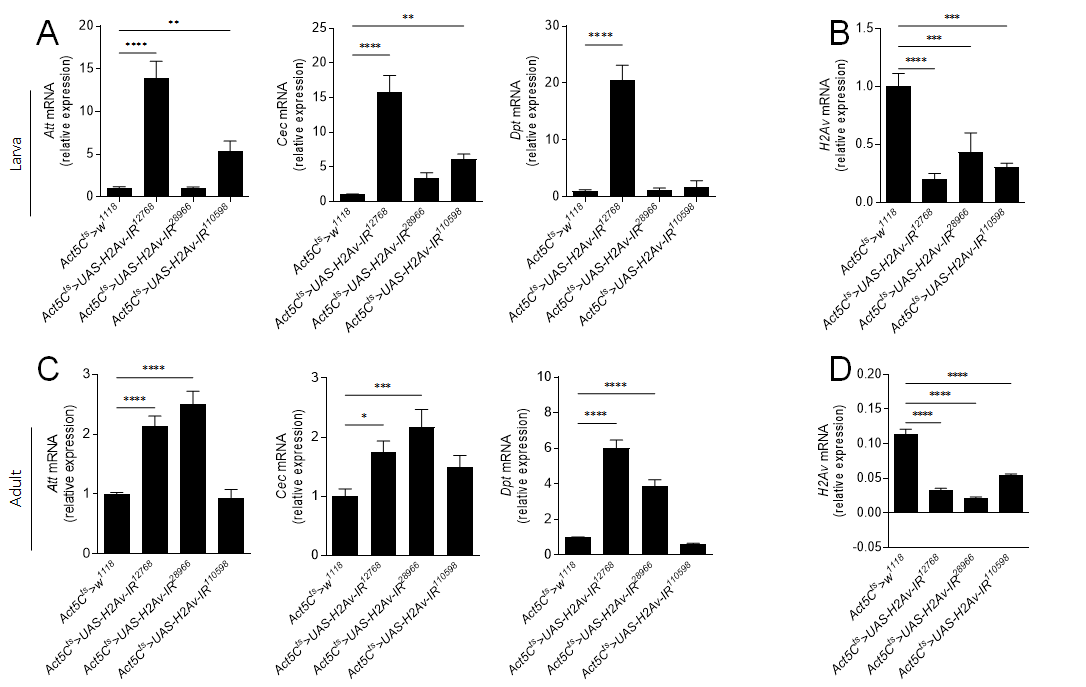

Supplement: S4 Fig — Three RNAi lines of H2Av were separately driven by Act5Cts and reared at 18°C. For larvae (A, B), offspring were brought to 29°C one day after hatching and fed for 2–3 d before dissecting fat bodies. For adults (C, D), offspring were brought to 29°C at day 3 after eclosion. Fat bodies were dissected and collected 3 d later. qPCR analysis of different AMPs was performed. The efficiency of knock-down of H2Av in larvae (B) and adults (D) was also analyzed. Knockdown of H2Av in either larvae or adults can significantly increase AMP production. One way ANOVA with Tukey’s multiple comparisons test was performed. Data represent the average of at least three independent assays (mean ± SE). *p < 0.05, **p < 0.01, ***p < 0.001, and ****p < 0.0001. (TIF) [file pgen.1009718.s004.tif]

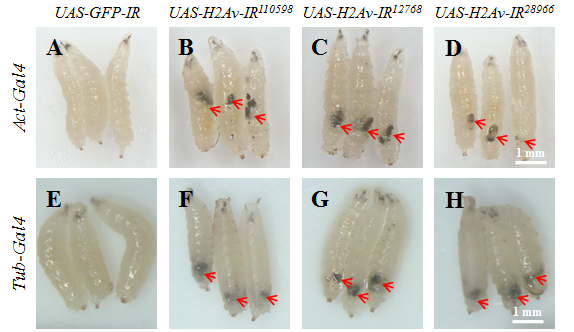

Supplement: S5 Fig — Three RNAi lines of H2Av were driven by Act-gal4 or Tub-gal4. Melanotic tumors as arrows indicated were observed near the posterior end of the larvae, a phenotype which is similar to H2Av810/H2Av810 mutant larvae. Knockdown of GFP was the control. Bar: (A-D) and (E-H) 1 mm. (TIF) [file pgen.1009718.s005.tif]

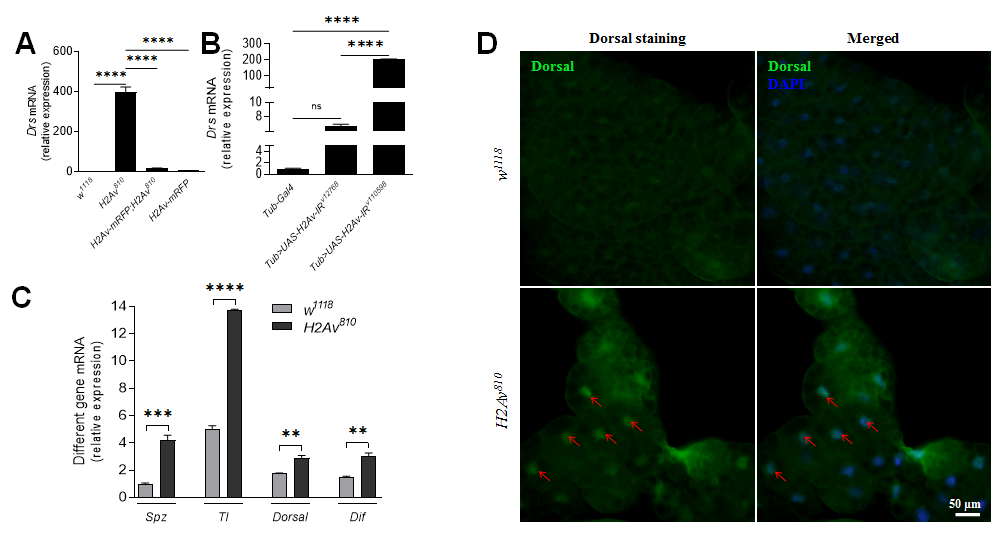

Supplement: S6 Fig — (A) Drs was significantly up-regulated in H2Av810 mutant larvae, which can be rescued by expression of H2Av-mRFP. (B) Knock-down of H2Av enhances the production of Drs in larvae. Two RNAi lines of H2Av were driven by Tub-gal4. qPCR analysis shows significant upregulation of Drs in the fat bodies after RNAi. (C) Up-regulation of Toll pathway components in H2Av810 mutant larvae. Fat bodies of H2Av810 mutant and w1118 second-instar larvae were dissected for qPCR analysis. Although the results show that main Toll pathway genes are upregulated, further study is needed to determine the role of the Toll pathway since Drs is synergistically regulated by the IMD and Toll pathways [2,37,38]. Data represent the average of at least three independent assays (mean ± SE) in (A-C). (D) Nuclear translocation of Dorsal in the fat bodies of H2Av810 mutant larvae. Dorsal and Dif are transcription factors of the Toll pathway that will translocate into the nuclei if this pathway is activated. Compared with w1118, Dorsal signal was detected in the nuclei of fat bodies of H2Av810 mutant larvae (arrow-indicated). One way ANOVA with Tukey’s multiple comparisons test (A, B) or two-tailed Student’s t-test (C) was performed. **p < 0.01, ***p < 0.001, and ****p < 0.0001. Bar: 50 μm. (TIF) [file pgen.1009718.s006.tif]

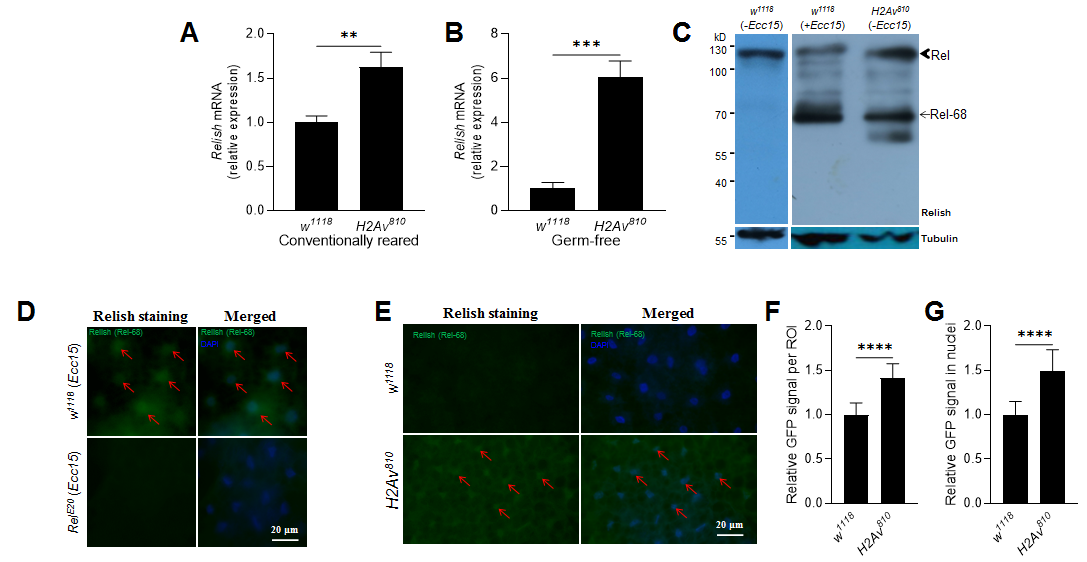

Supplement: S7 Fig — (A, B) qPCR analysis of Relish in the fat bodies of larvae conventionally reared (A) or germ-free (B) before the wandering stage. (C) Western blotting shows Relish and Rel-68 in w1118 (received Ecc15 challenge or not as a positive or negative control) and H2Av810 mutants (without Ecc15 challenge). Polyclonal antiserum against Drosophila Relish [40] was used for the Western blotting assay and active Rel-68 was detected in H2Av810. The arrowhead and arrow point to Relish (Rel) and Rel-68 respectively. (D) Commercial antibody against Drosophila Relish (Abin1111036, RayBiotech 130–10080) was applied for immuno-staining fat bodies dissected from w1118 and RelE20 received immune challenged according to the published papers [41,42]. Using this commercial antibody, we detected Relish signal in nuclei of fat bodies of w1118 but not RelE20 mutant larvae. (E) Immuno-staining to show the distribution of Relish in fat body cells and Rel-68 in nuclei of larval fat bodies of H2Av810 mutant. The arrows indicate cells with Relish signal inside nuclei. (F-G) Quantification of Relish in whole fat body cells (F) and Rel-68 in nuclei (G). The amount of Rel or Rel-68 produced from fat bodies of w1118 is equal to 1 for quantification. Two-tailed Student’s t-test was performed. Mean values are presented ±SE. **p < 0.01, ***p < 0.001, and ****p < 0.0001. Bar: 20 μm. (TIF) [file pgen.1009718.s007.tif]

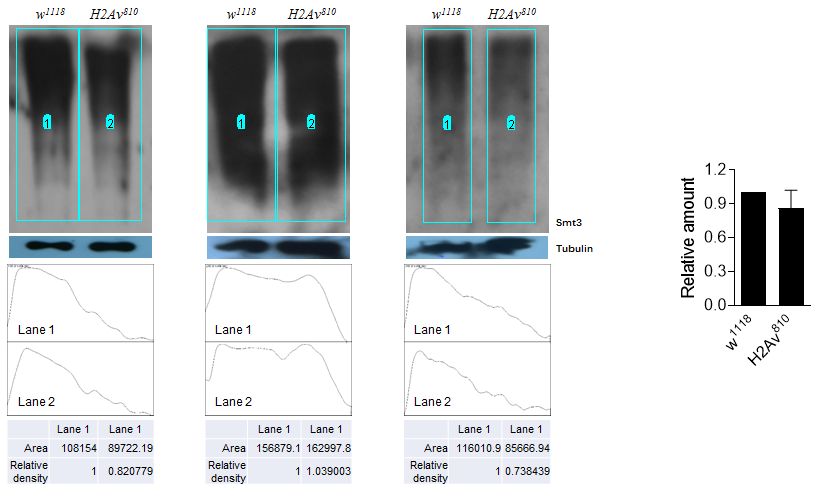

Supplement: S8 Fig — Proteins lysed from fat bodies of three larvae were loaded for each lane. The polyclonal antibody against Smt3 used in this study can detect SUMOylated proteins. Each blot was quantified using ImageJ from the NIH (https://imagej.nih.gov/ij/docs/guide/146-30.html). The lanes were plotted as indicated for quantification. The histogram of each lane was also placed under the corresponding blot. The area of each peak was enclosed for calculating the area that was listed below. Each w1118 = 1, and the relative density was calculated. This experiment was repeated 3 times independently. (TIF) [file pgen.1009718.s008.tif]

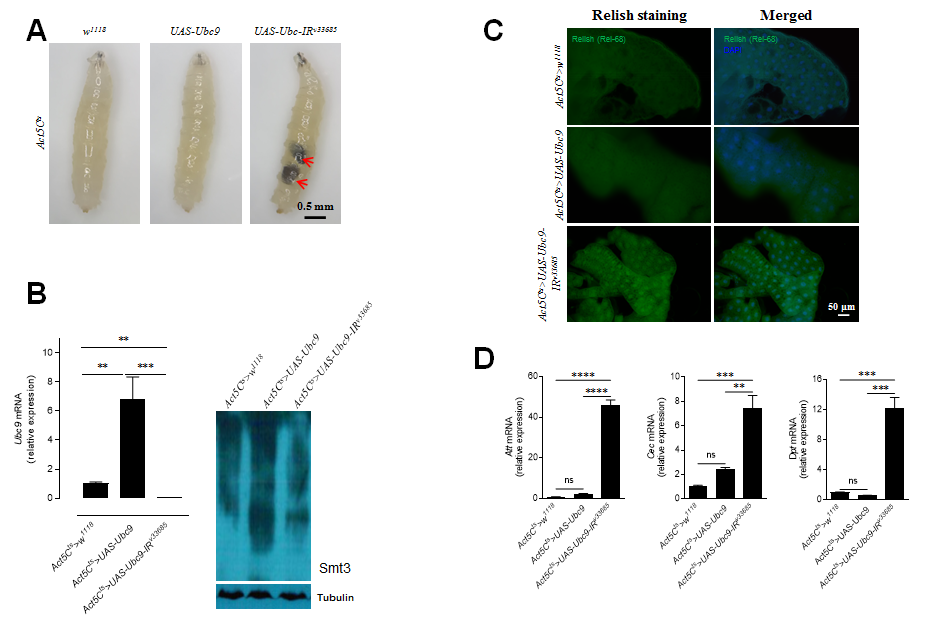

Supplement: S9 Fig — (A) Knockdown of Ubc9 produces melanotic tumors in larvae. Ubc9 was knocked down or over-expressed via Act5Cts. Melanotic tumors, as arrows indicate, were observed near the posterior end of Ubc9 knockdown larvae but not the control and Ubc9 over-expression larvae, a phenotype which is similar to H2Av810/H2Av810 mutant larvae. (B) qPCR and western blot to show the transcription level of Ubc9 and the SUMOylated proteins after knockdown or over-expression of Ubc9. Data represent the average of three independent assays (mean ± SE). (C) Commercial antibody against Drosophila Relish (Abin1111036, RayBiotech 130–10080) was applied for the immuno-staining of fat bodies dissected from the above larvae that received no immunochallenge. Relish signal was detected in nuclei of fat bodies of Ubc9 knockdown larvae but not the control and Ubc9 over-expression larvae. (D) In Ubc9 knockdown larvae but not the control and Ubc9 over-expression larvae the production of AMPs was enhanced. Data represent the average of three independent assays (mean ± SE). One way ANOVA with Tukey’s multiple comparisons test was performed. **p < 0.01 and ***p < 0.001. Bar: (A) 0.5 mm. (B) 50 μm. (TIF) [file pgen.1009718.s009.tif]

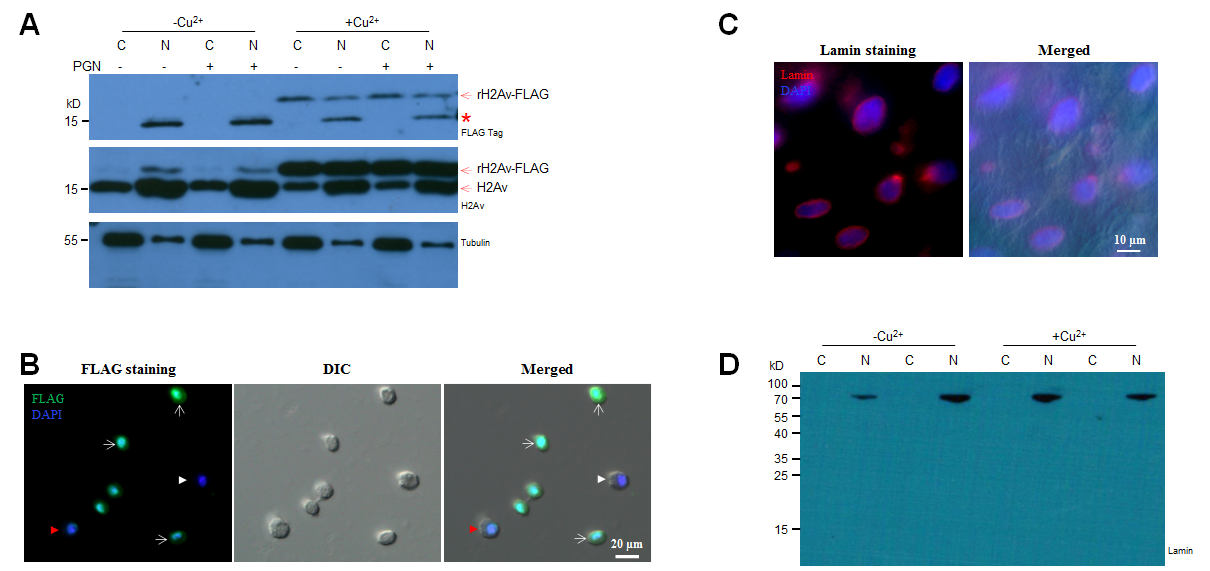

Supplement: S10 Fig — (A) Recombinant H2Av-Flag (rH2Av-Flag) was transfected into S2 cells to make a stable cell line using the Cu2+ inducible pMT/V5-His vector as described [56]. S2 cells stimulated or not to express rH2Av-Flag were lysed and the cytoplasmic (C) and nuclear (N) fractions were separated. Western blots using antibody against Flag-tag and H2Av. * indicate that one nuclei protein was non-specifically detected by the FLAG antibody. Both endogenous H2Av and newly-expressed rH2Av-Flag were detected in the cytoplasm and nuclei. (B) Detection of rH2Av-Flag in cytoplasm and nuclei. When rH2Av-Flag was induced to express, S2 cells were fixed for immuno-staining and observed using confocal microscopy. The arrows indicate Flag-staining signal in the cytoplasm and nuclei of some S2 cells. The red arrowhead indicates the Flag signal in the nucleus but not in the cytoplasm, which might be due to the low amount of rH2Av-Flag expressed. The white arrowhead indicates one S2 cell without rH2Av-Flag expression due to no Flag signal staining. The immuno-staining also demonstrates that there is cytoplasmic H2Av in Drosophila cells. (C) Detection of lamin in the nuclei of adult midguts. Lamin is a nuclei-associated protein and the anti-lamin antibody used for immuno-staining showed its location around the nuclei of midgut cells. (D) Detection of lamin in the cytoplasmic and nuclear fractions. There was no lamin signal in the cytoplasmic fraction as shown in (A), indicating no contamination of nuclear proteins during separation. (TIF) [file pgen.1009718.s010.tif]

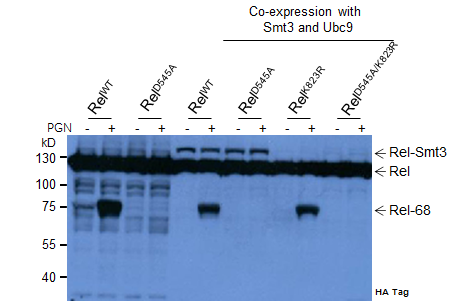

Supplement: S11 Fig — To avoid Relish auto-activation [18] which may interfere with the influence of Relish SUMOylation on cleavage, cells were collected after being transiently transfected for 30 h. Relish is cleaved at D545 for activation [19]. When D545 was mutated (RelD545A) and over-expressed in S2 cells, it could not be cleaved following PGN stimulation for 2 h. However, SUMOylation was not affected. K823 is the main site for SUMOylation. When mutant RelK823R (loss of SUMOylation) was over-expressed in S2 cells, it could be cleaved after PGN stimulation. Double mutant RelD545A/K823R could neither be SUMOylated nor cleaved. The arrows indicate the positions of Rel-Smt3, Rel (wild type and different mutants) and Rel-68 respectively. (TIF) [file pgen.1009718.s011.tif]

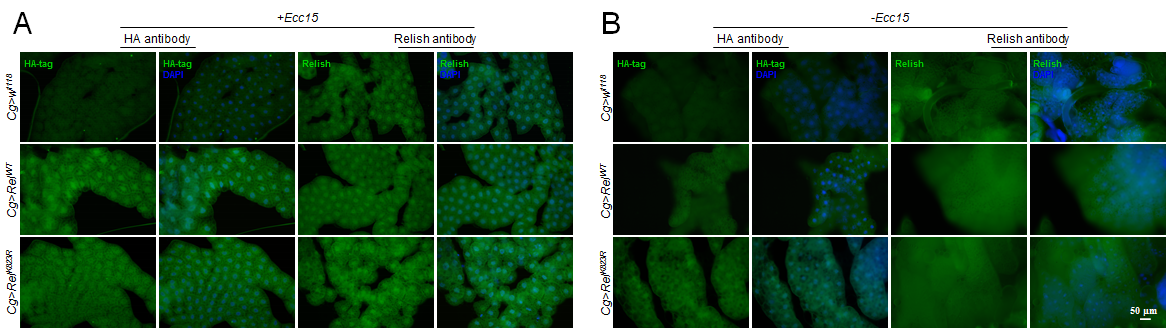

Supplement: S12 Fig — Fat bodies from larvae that received Ecc15 injection (A) or not (B) were fixed for Relish immuno-staining. For fat bodies from HA-RelWT–V5/His and HA-RelK823R-V5/His over-expressed larvae, if Ecc15 was not injected, no signal of native Relish was detected inside nuclei using the commercial antibody against Relish [41,42]. (TIF) [file pgen.1009718.s012.tif]

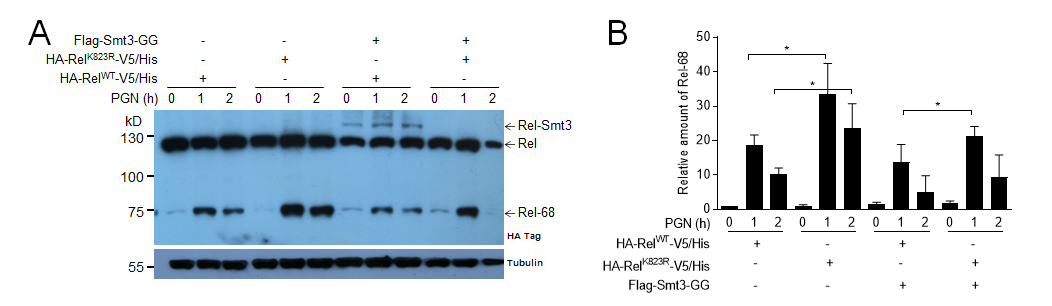

Supplement: S13 Fig — HA-RelWT-V5/His and HA-RelK823R-V5/His (loss of main site for SUMOylation) were separately co-expressed with Smt3-GG or not as indicated for 48 h (A), PGN was applied for different periods. Western blot shows the cleavage of RelWT and RelK823R. The arrows indicate the positions of Rel-Smt3, Rel (RelWT and RelK823R) and Rel-68 separately. The production of Rel-68 was quantified for each treatment (B). Loss of potential SUMOylation can enhance Relish cleavage. Data represent the average of three independent assays (mean ± SE). Two-tailed Student’s t-test was performed. *p < 0.05. (TIF) [file pgen.1009718.s013.tif]

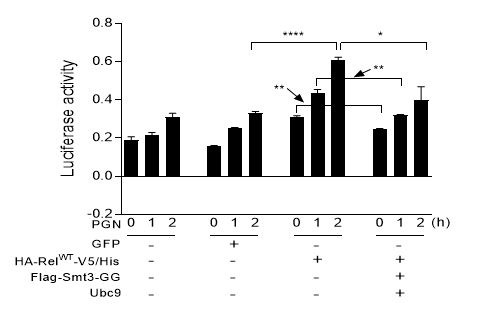

Supplement: S14 Fig — Reporter assays using the luciferase gene under the control of the Cecropin promoter. GFP or RelWT alone were over-expressed with the above reporter system. RelWT, Smt3-GG and Ube 9 were co-expressed with the above reporter system for 48 h. Then PGN was applied to induce Relish activation and cleavage. At each indicated time point, significantly higher luciferase activities were detected in the treatment without obvious SUMOylation (RelWT alone) compared to those with SUMOylation (RelWT, Smt3-GG and Ubc9 co-expressed). Data represent the average of three independent assays (mean ± SE). Two-tailed Student’s t-test was performed. *p < 0.05, **p < 0.01, and ****p < 0.0001. (TIF) [file pgen.1009718.s014.tif]

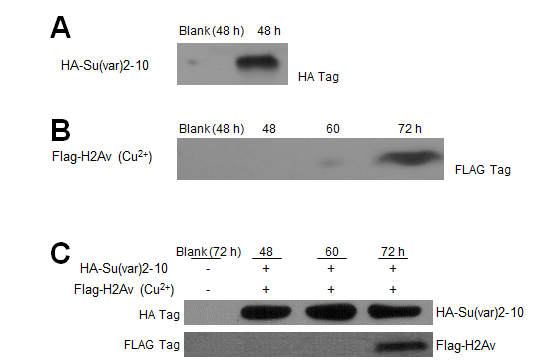

Supplement: S15 Fig — The sequences of Flag-H2Av and HA-Su(Var)2-10 were inserted into the pMT/V5-His vector respectively for transient expression in S2 cells. The same number of S2 cells were collected and lysed in the same volume of 1X SDS loading buffer at each time point. HA-Su(Var)2-10 was expressed in a large amount at 48 h as detected using a Western blot assay (A). There was almost no expression of Flag-H2Av at 48 h until 60–72 h (B). Transient co-expression of Flag-H2Av and HA-Su(Var)2-10 did interrupt each other (C). Therefore, the expression of Flag-H2Av lags behind that of HA-Su(Var)2-10 for an unknown reason. Thus, a stable cell line to express Flag-H2Av was constructed for in vitro experiments. Blank: S2 cells were transiently transfected with the same amount of pMT/V5-His vector without any gene inserted. (TIF) [file pgen.1009718.s015.tif]

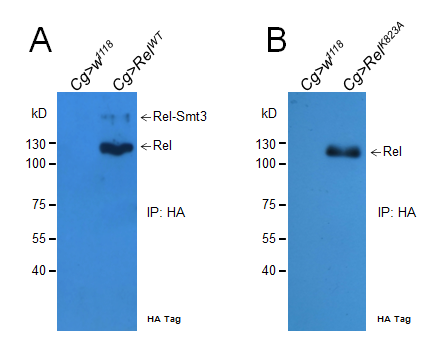

Supplement: S16 Fig — HA-RelWT-V5/His (A) or HA-RelK823R-V5/His (B) was over-expressed in larval fat bodies using Cg-gal4. SUMOylated Relish was enriched using antibody against HA-tag conjugated beads. Fat bodies from 50 larvae were collected and lysed for pull-down and antibody against the HA-tag was used in a Western blot to detect the SUMOylated Relish. The arrows indicate Rel-Smt3 and Rel respectively. In vivo, SUMOylated HA-RelWT-V5/His-Smt3 (Rel-Smt3) but not HA-RelK823R-V5/His-Smt3 was detected but the amount was low. (TIF) [file pgen.1009718.s016.tif]
